# Supplementary material for: Arterial spin labeling versus BOLD in direct challenge and drug-task interaction pharmacological fMRI
Source: PeerJ. 2014 Dec 11;2:e687. doi: 10.7717/peerj.687 (PMC4266850; doi:10.7717/peerj.687)
Supplement: Supplemental Information 1 [file peerj-02-687-s001.pdf]

SYN increases fixand2back, 12 subs, 5p7mm

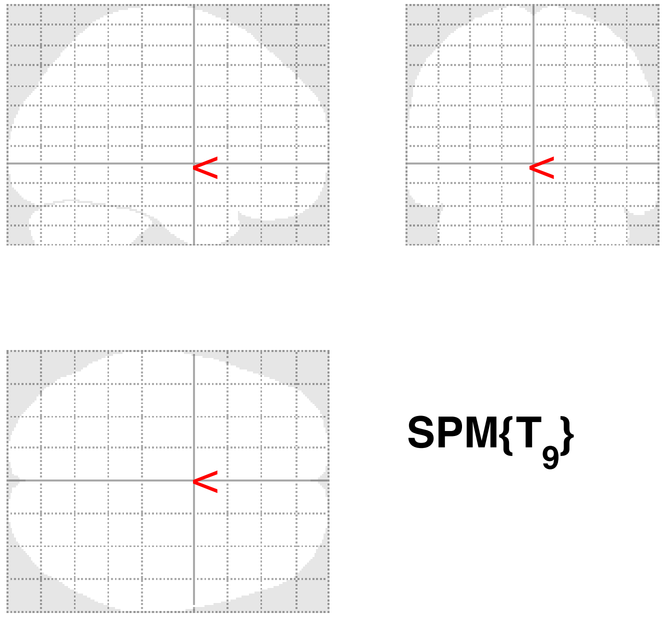

SPM{T_9}

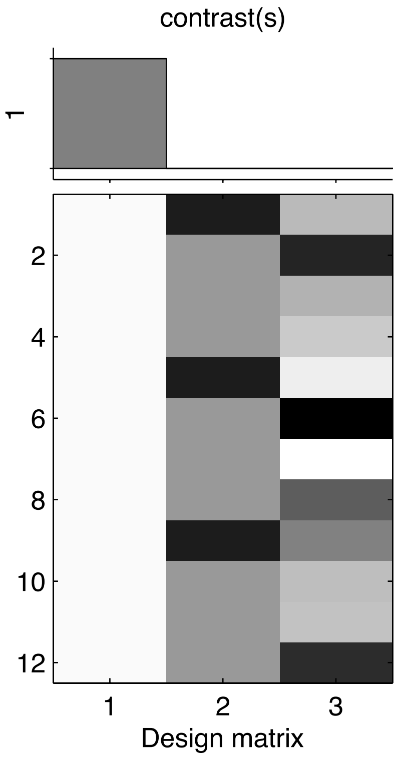

SPMresults: ixand2back_12subs_only_5p7mm
Height threshold T = 4.296806 {p<0.001 (unc.)}
Extent threshold k = 0 voxels

Statistics: ***p−values adjusted for search volume***

set−level		cluster−level				peak−level					mm mm mm
p	c	$p_{FWE-corr}$	$q_{FDR-corr}$	k_E	p_{uncorr}	$p_{FWE-corr}$	$q_{FDR-corr}$	T	$(Z_{\equiv})$	p_{uncorr}	

no suprathreshold clusters

table shows 3 local maxima more than 8.0mm apart

Height threshold: T = 4.30, p = 0.001 (1.000)
Extent threshold: k = 0 voxels
Expected voxels per cluster, <k> = 1.863
Expected number of clusters, <c> = 26.75
FWEp: 10.666, FDRp: Inf, FWEc: Inf, FDRc: Inf

Degrees of freedom = [1.0, 9.0]
FWHM = 9.3 11.0 10.2 mm mm mm; 3.1 3.7 3.4 {voxels}
Volume: 1294110 = 47930 voxels = 1111.9 resels
Voxel size: 3.0 3.0 3.0 mm mm mm; (resel = 38.73 voxels)

SYN decreases, fixand2back, 12 subs only, 5p7m

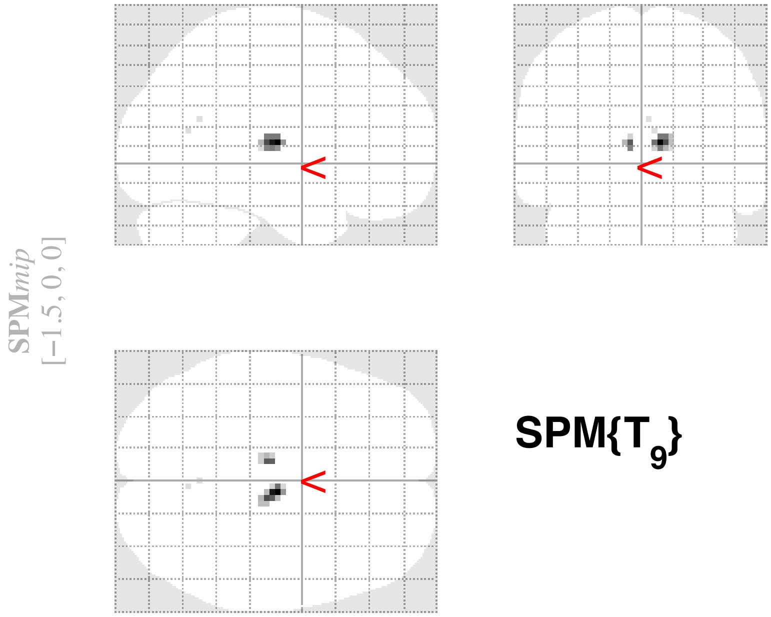

SPM{T_9}

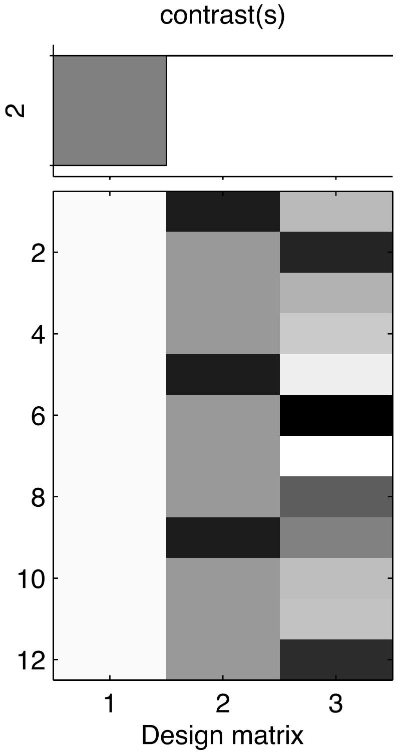

SPMresults:ixand2back_12subs_only_5p7mm
Height threshold T = 4.296806 {p<0.001 (unc.)}
Extent threshold k = 0 voxels

Statistics: *p-values adjusted for search volume*

set-level		cluster-level				peak-level					mm mm mm
p	c	$p_{FWE-corr}$	$q_{FDR-corr}$	k_E	p_{uncorr}	$p_{FWE-corr}$	$q_{FDR-corr}$	T	$(Z_{\equiv})$	p_{uncorr}	
1.000	4	0.029	0.004	25	0.001	1.000	0.993	5.81	3.66	0.000	8 −15 9
		0.482	0.049	10	0.025	1.000	0.993	5.17	3.44	0.000	−8 −21 9
		1.000	0.450	1	0.450	1.000	0.993	4.31	3.09	0.001	4 −63 15
		1.000	0.450	1	0.450	1.000	0.993	4.31	3.09	0.001	2 −57 21

table shows 3 local maxima more than 8.0mm apart

Height threshold: T = 4.30, p = 0.001 (1.000)
Extent threshold: k = 0 voxels
Expected voxels per cluster, <k> = 1.863
Expected number of clusters, <c> = 26.75
FWEp: 10.666, FDRp: Inf, FWEc: 25, FDRc: 10

Degrees of freedom = [1.0, 9.0]
FWHM = 9.3 11.0 10.2 mm mm mm; 3.1 3.7 3.4 {voxels}
Volume: 1294110 = 47930 voxels = 1111.9 resels
Voxel size: 3.0 3.0 3.0 mm mm mm; (resel = 38.73 voxels)
