# Supplementary material for: Arterial spin labeling versus BOLD in direct challenge and drug-task interaction pharmacological fMRI
Source: PeerJ. 2014 Dec 11;2:e687. doi: 10.7717/peerj.687 (PMC4266850; doi:10.7717/peerj.687)
Supplement: Supplemental Information 2 [file peerj-02-687-s002.pdf]

BOLD SYN increases, 60 mg

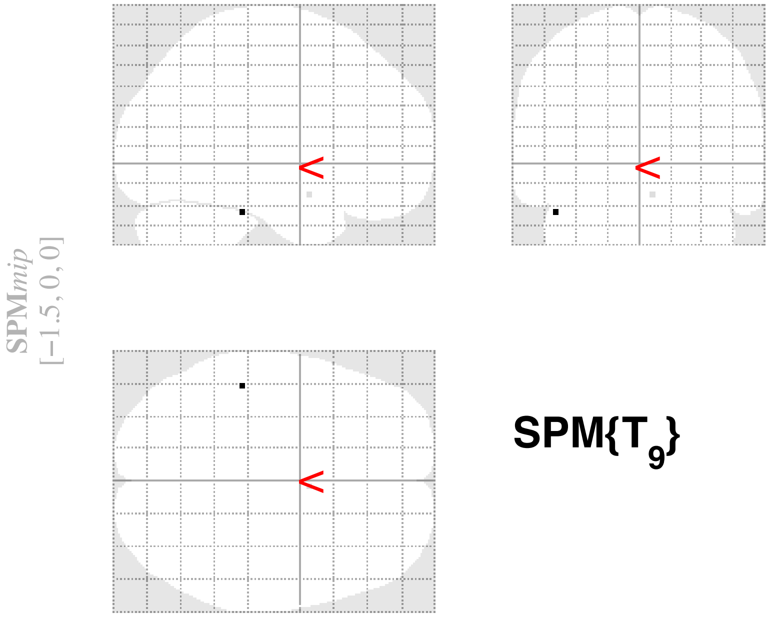

SPM{T_9}

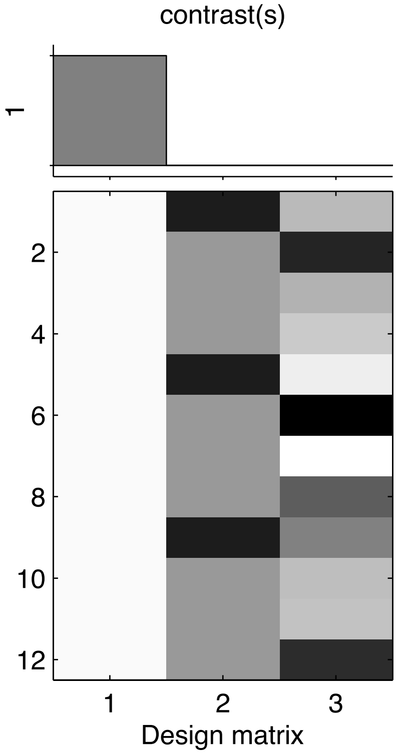

SPMresults: /bold_SYN_effect_60_only
Height threshold T = 4.296806 {p<0.001 (unc.)}
Extent threshold k = 0 voxels

Statistics: ***p*–values adjusted for search volume**

set–level		cluster–level				peak–level					mm mm mm
p	c	$p_{FWE-corr}$	$q_{FDR-corr}$	k_E	p_{uncorr}	$p_{FWE-corr}$	$q_{FDR-corr}$	T	$(Z_{\equiv})$	p_{uncorr}	
1.000	2	1.000	0.397	1	0.397	1.000	0.595	5.10	3.41	0.000	−46 −33 −27
		1.000	0.397	1	0.397	1.000	0.595	4.96	3.36	0.000	4 3 −18

table shows 3 local maxima more than 8.0mm apart

Height threshold: T = 4.30, p = 0.001 (1.000)
Extent threshold: k = 0 voxels
Expected voxels per cluster, <k> = 1.498
Expected number of clusters, <c> = 42.98
FWEp: 11.012, FDRp: Inf, FWEc: Inf, FDRc: Inf

Degrees of freedom = [1.0, 9.0]
FWHM = 10.0 10.2 8.2 mm mm mm; 3.3 3.4 2.7 {voxels}
Volume: 1692981 = 62703 voxels = 1822.3 resels
Voxel size: 3.0 3.0 3.0 mm mm mm; (resel = 31.15 voxels)

BOLD SYN decreases 60 mg only

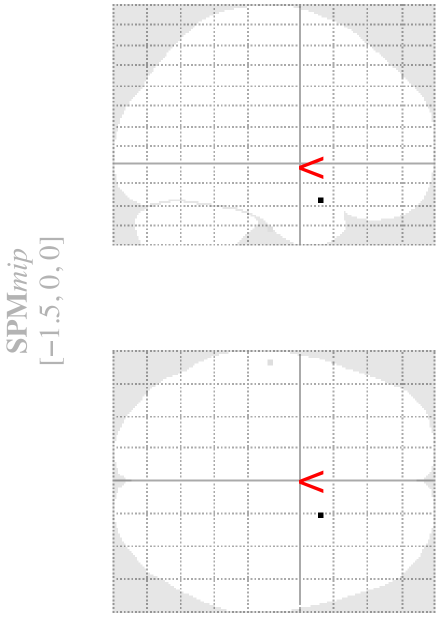

SPM{T_9}

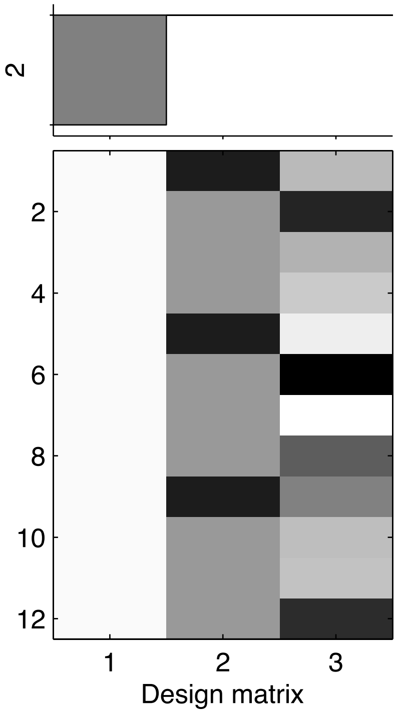

SPMresults: /bold_SYN_effect_60_only
Height threshold T = 4.296806 {p<0.001 (unc.)}
Extent threshold k = 0 voxels

Statistics: ***p−values adjusted for search volume***

set−level		cluster−level				peak−level					mm mm mm		
p	c	$p_{FWE-corr}$	$q_{FDR-corr}$	k_E	p_{uncorr}	$p_{FWE-corr}$	$q_{FDR-corr}$	T	$(Z_\equiv)$	p_{uncorr}			
1.000	2	1.000	0.397	1	0.397	1.000	0.549	5.97	3.71	0.000	20	9	−21
		1.000	0.397	1	0.397	1.000	0.820	4.55	3.20	0.001	−58	−18	−36

table shows 3 local maxima more than 8.0mm apart

Height threshold: T = 4.30, p = 0.001 (1.000)
Extent threshold: k = 0 voxels
Expected voxels per cluster, <k> = 1.498
Expected number of clusters, <c> = 42.98
FWEp: 11.012, FDRp: Inf, FWEc: Inf, FDRc: Inf

Degrees of freedom = [1.0, 9.0]
FWHM = 10.0 10.2 8.2 mm mm mm; 3.3 3.4 2.7 {voxels}
Volume: 1692981 = 62703 voxels = 1822.3 resels
Voxel size: 3.0 3.0 3.0 mm mm mm; (resel = 31.15 voxels)
