# Supplementary material for: Arterial spin labeling versus BOLD in direct challenge and drug-task interaction pharmacological fMRI
Source: PeerJ. 2014 Dec 11;2:e687. doi: 10.7717/peerj.687 (PMC4266850; doi:10.7717/peerj.687)
Supplement: Supplemental Information 3 [file peerj-02-687-s003.pdf]

2 back increases 60 mg only 5p7mm

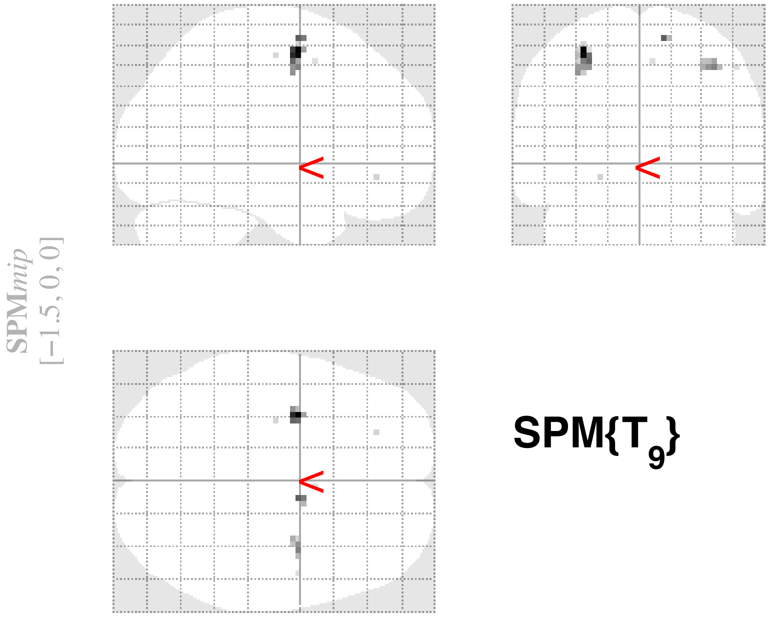

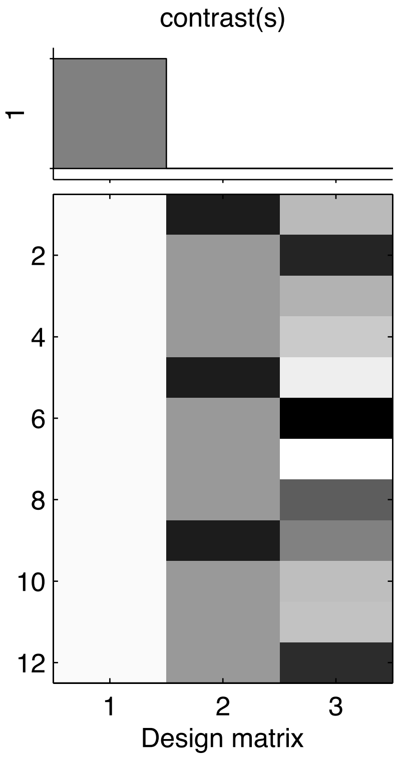

SPMresults: back_effects_60mg_only_5p7mm
Height threshold T = 4.296806 {p<0.001 (unc.)}
Extent threshold k = 0 voxels

Statistics: *p-values adjusted for search volume*

set-level		cluster-level				peak-level					mm mm mm		
p	c	$p_{FWE-corr}$	$q_{FDR-corr}$	k_E	p_{uncorr}	$p_{FWE-corr}$	$q_{FDR-corr}$	T	$(Z_{\equiv})$	p_{uncorr}			
1.000	7	0.090	0.030	22	0.004	0.998	0.968	5.88	3.68	0.000	-32	-3	57
						1.000	0.968	4.84	3.32	0.000	-34	-6	45
		0.994	0.500	3	0.236	1.000	0.968	5.20	3.45	0.000	10	-3	63
		0.664	0.174	9	0.050	1.000	0.968	4.97	3.37	0.000	38	-3	48
		1.000	0.500	1	0.500	1.000	0.968	4.41	3.14	0.001	-22	39	-9
		1.000	0.500	1	0.500	1.000	0.968	4.40	3.13	0.001	-28	-15	54
		1.000	0.500	1	0.500	1.000	0.968	4.35	3.11	0.001	4	6	51
		1.000	0.500	1	0.500	1.000	0.968	4.34	3.11	0.001	50	-3	48

table shows 3 local maxima more than 8.0mm apart

Height threshold: T = 4.30, p = 0.001 (1.000)
Extent threshold: k = 0 voxels
Expected voxels per cluster, <k> = 2.302
Expected number of clusters, <c> = 21.92
FWEp: 10.666, FDRp: Inf, FWEc: Inf, FDRc: 22

Degrees of freedom = [1.0, 9.0]
FWHM = 9.8 11.1 12.0 mm mm mm; 3.3 3.7 4.0 {voxels}
Volume: 1294110 = 47930 voxels = 900.0 resels
Voxel size: 3.0 3.0 3.0 mm mm mm; (resel = 47.85 voxels)

2 back decreases 60 mg only 5p7mm

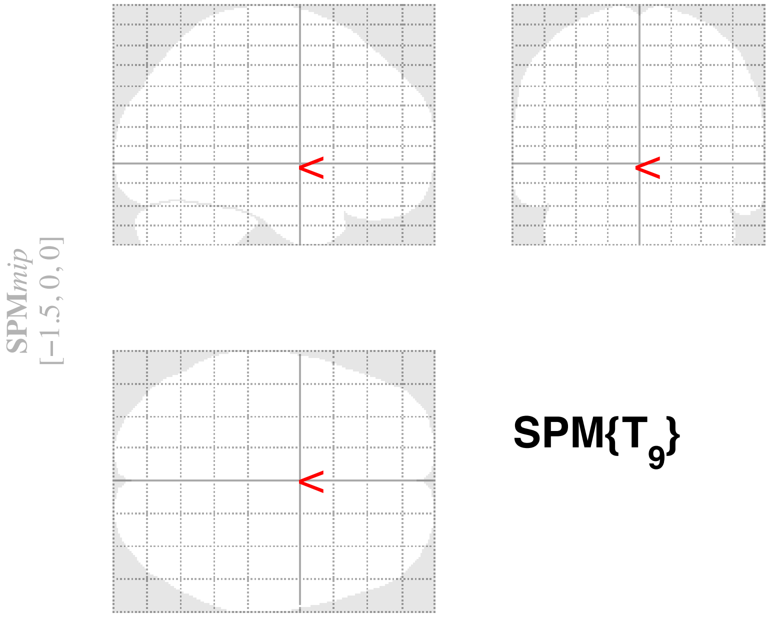

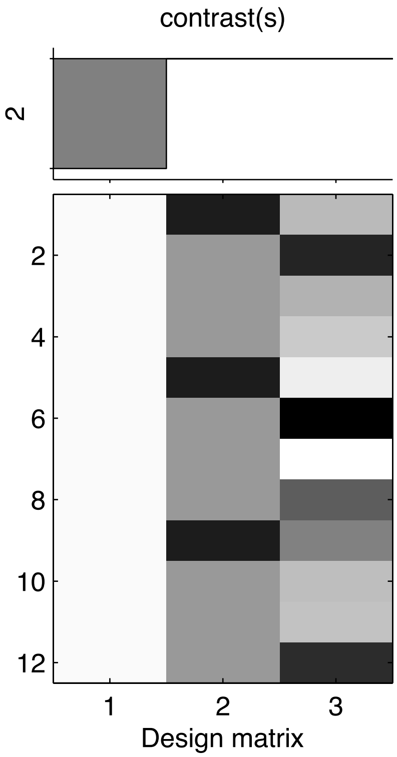

SPM{T_9}

SPMresults: back_effects_60mg_only_5p7mm
Height threshold T = 4.296806 {p<0.001 (unc.)}
Extent threshold k = 0 voxels

Statistics: ***p−values adjusted for search volume***

set−level		cluster−level				peak−level					mm mm mm
p	c	$p_{FWE-corr}$	$q_{FDR-corr}$	k_E	p_{uncorr}	$p_{FWE-corr}$	$q_{FDR-corr}$	T	($Z_\equiv$)	p_{uncorr}	

no suprathreshold clusters

table shows 3 local maxima more than 8.0mm apart

Height threshold: T = 4.30, p = 0.001 (1.000)
Extent threshold: k = 0 voxels
Expected voxels per cluster, <k> = 2.302
Expected number of clusters, <c> = 21.92
FWEp: 10.666, FDRp: Inf, FWEc: Inf, FDRc: Inf

Degrees of freedom = [1.0, 9.0]
FWHM = 9.8 11.1 12.0 mm mm mm; 3.3 3.7 4.0 {voxels}
Volume: 1294110 = 47930 voxels = 900.0 resels
Voxel size: 3.0 3.0 3.0 mm mm mm; (resel = 47.85 voxels)
