# Supplementary material for: Arterial spin labeling versus BOLD in direct challenge and drug-task interaction pharmacological fMRI
Source: PeerJ. 2014 Dec 11;2:e687. doi: 10.7717/peerj.687 (PMC4266850; doi:10.7717/peerj.687)
Supplement: Supplemental Information 5 [file peerj-02-687-s005.pdf]

SYN x 2back increases, 60 mg only, 5p7mm

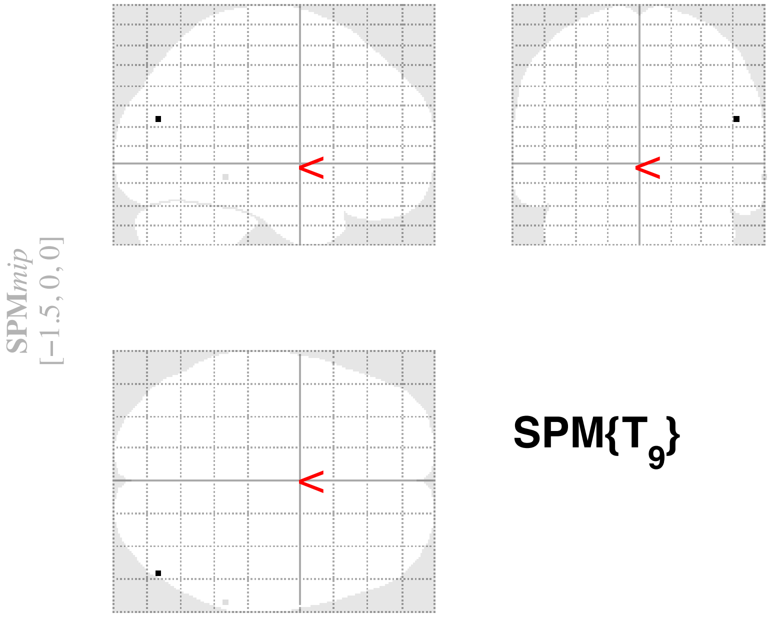

SPM{T_9}

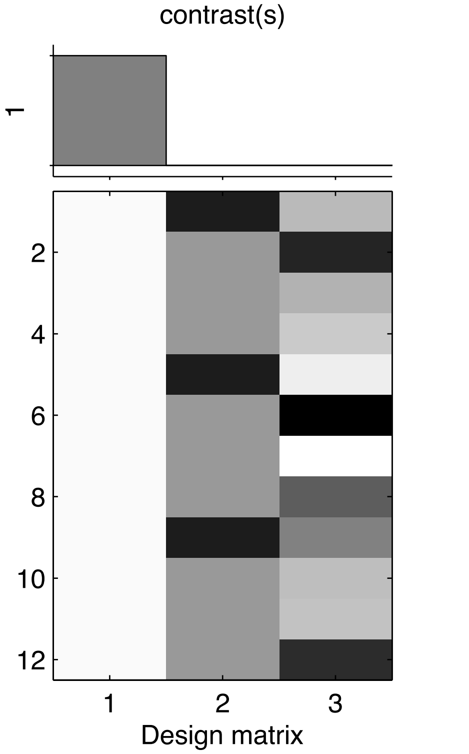

SPMresults: /SYNx2back_60mg_only_5p7mm
Height threshold T = 4.296806 {p<0.001 (unc.)}
Extent threshold k = 0 voxels

Statistics: ***p-values adjusted for search volume***

set-level		cluster-level				peak-level					mm mm mm
p	c	$p_{FWE-corr}$	$q_{FDR-corr}$	k_E	p_{uncorr}	$p_{FWE-corr}$	$q_{FDR-corr}$	T	$(Z_{\equiv})$	p_{uncorr}	
1.000	2	1.000	0.449	1	0.449	1.000	0.871	4.93	3.35	0.000	50 −78 21
		1.000	0.449	1	0.449	1.000	0.871	4.47	3.16	0.001	64 −42 −9

table shows 3 local maxima more than 8.0mm apart

Height threshold: T = 4.30, p = 0.001 (1.000)
Extent threshold: k = 0 voxels
Expected voxels per cluster, <k> = 1.852
Expected number of clusters, <c> = 26.94
FWEp: 10.666, FDRp: Inf, FWEc: Inf, FDRc: Inf

Degrees of freedom = [1.0, 9.0]
FWHM = 9.3 10.4 10.8 mm mm mm; 3.1 3.5 3.6 {voxels}
Volume: 1294110 = 47930 voxels = 1118.6 resels
Voxel size: 3.0 3.0 3.0 mm mm mm; (resel = 38.50 voxels)

SYN x 2 back decreases, 60 mg only, 5p7mm

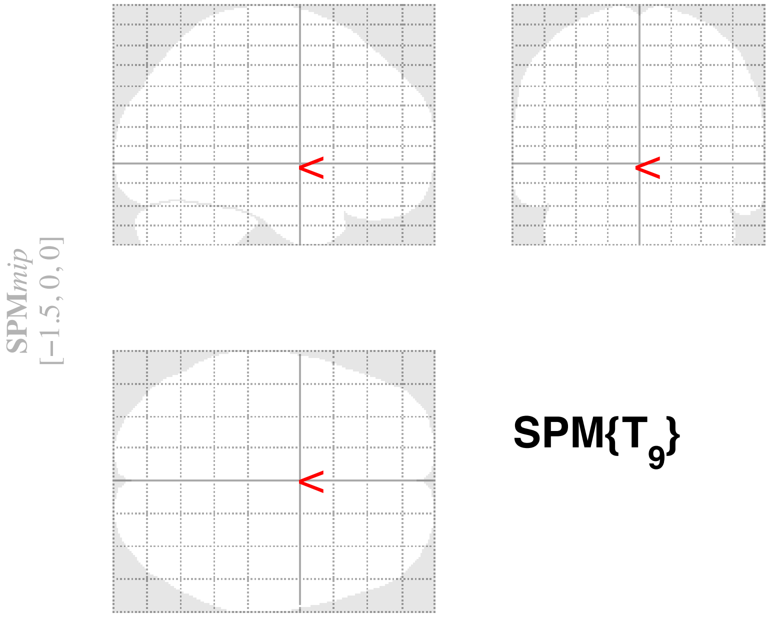

SPM{T_9}

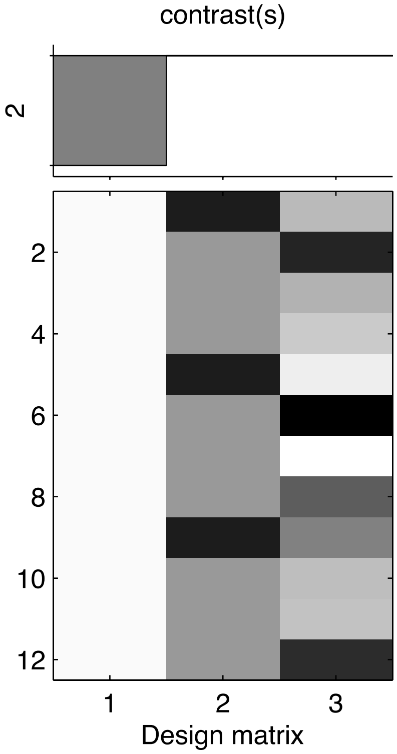

SPMresults: /SYNx2back_60mg_only_5p7mm
Height threshold T = 4.296806 {p<0.001 (unc.)}
Extent threshold k = 0 voxels

Statistics: ***p−values adjusted for search volume***

set−level		cluster−level				peak−level					mm mm mm
p	c	$p_{FWE-corr}$	$q_{FDR-corr}$	k_E	p_{uncorr}	$p_{FWE-corr}$	$q_{FDR-corr}$	T	$(Z_{\equiv})$	p_{uncorr}	

no suprathreshold clusters

table shows 3 local maxima more than 8.0mm apart

Height threshold: T = 4.30, p = 0.001 (1.000)
Extent threshold: k = 0 voxels
Expected voxels per cluster, <k> = 1.852
Expected number of clusters, <c> = 26.94
FWEp: 10.666, FDRp: Inf, FWEc: Inf, FDRc: Inf

Degrees of freedom = [1.0, 9.0]
FWHM = 9.3 10.4 10.8 mm mm mm; 3.1 3.5 3.6 {voxels}
Volume: 1294110 = 47930 voxels = 1118.6 resels
Voxel size: 3.0 3.0 3.0 mm mm mm; (resel = 38.50 voxels)
