# Supplementary material for: Arterial spin labeling versus BOLD in direct challenge and drug-task interaction pharmacological fMRI
Source: PeerJ. 2014 Dec 11;2:e687. doi: 10.7717/peerj.687 (PMC4266850; doi:10.7717/peerj.687)
Supplement: Supplemental Information 6 [file peerj-02-687-s006.pdf]

BOLD SYN on 2back increases 60 mg only

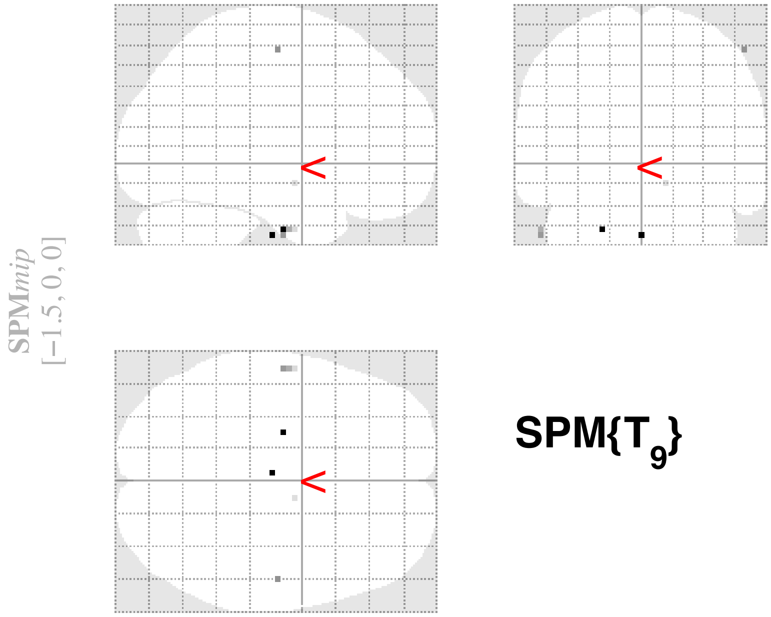

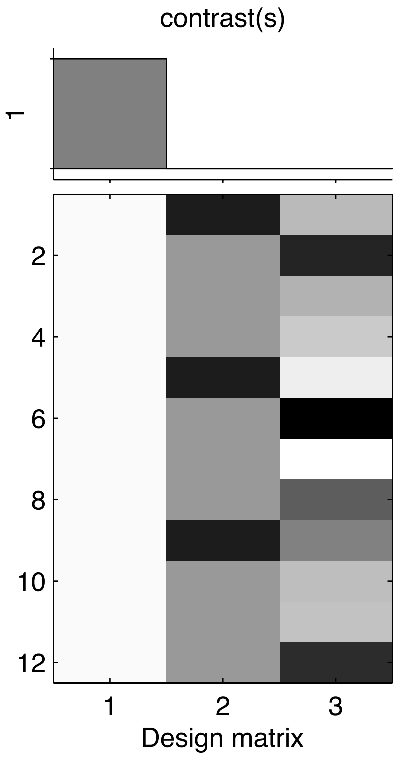

SPMresults: /bold_SYN_on_2back_60_only
Height threshold T = 4.296806 {p<0.001 (unc.)}
Extent threshold k = 0 voxels

Statistics: ***p−values adjusted for search volume***

set−level		cluster−level				peak−level					mm mm mm
p	c	$p_{FWE-corr}$	$q_{FDR-corr}$	k_E	p_{uncorr}	$p_{FWE-corr}$	$q_{FDR-corr}$	T	$(Z_{\equiv})$	p_{uncorr}	
1.000	5	1.000	0.382	1	0.382	1.000	0.892	5.09	3.41	0.000	−2 −18 −39
		1.000	0.382	1	0.382	1.000	0.892	5.07	3.40	0.000	−22 −12 −36
		1.000	0.382	1	0.382	1.000	0.892	4.66	3.24	0.001	52 −15 57
		0.998	0.382	3	0.135	1.000	0.892	4.64	3.23	0.001	−56 −12 −39
		1.000	0.382	1	0.382	1.000	0.892	4.44	3.15	0.001	10 −6 −12

table shows 3 local maxima more than 8.0mm apart

Height threshold: T = 4.30, p = 0.001 (1.000)
Extent threshold: k = 0 voxels
Expected voxels per cluster, <k> = 1.411
Expected number of clusters, <c> = 45.53
FWEp: 11.012, FDRp: Inf, FWEc: Inf, FDRc: Inf

Degrees of freedom = [1.0, 9.0]
FWHM = 9.5 10.0 8.3 mm mm mm; 3.2 3.3 2.8 {voxels}
Volume: 1692981 = 62703 voxels = 1935.4 resels
Voxel size: 3.0 3.0 3.0 mm mm mm; (resel = 29.33 voxels)

BOLD SYN on 2 back decreases 60 mg only

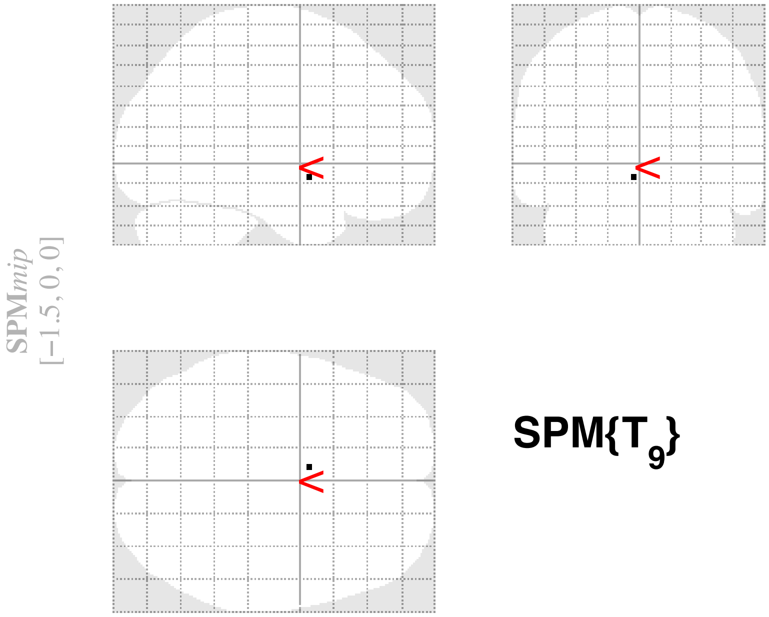

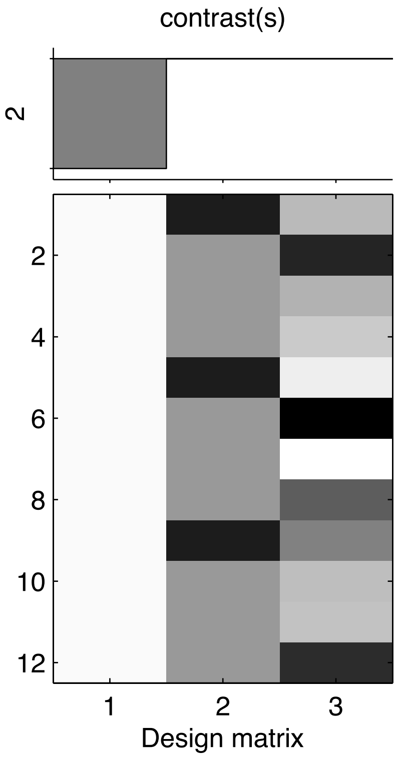

SPMresults: /bold_SYN_on_2back_60_only
Height threshold T = 4.296806 {p<0.001 (unc.)}
Extent threshold k = 0 voxels

Statistics: ***p-values adjusted for search volume***

	cluster-level				peak-level					mm mm mm
	$p_{FWE-corr}$	$q_{FDR-corr}$	k_E	p_{uncorr}	$p_{FWE-corr}$	$q_{FDR-corr}$	T	$(Z_{\equiv})$	p_{uncorr}	
	1.000	0.382	1	0.382	1.000	0.629	4.89	3.33	0.000	−4 3 −9

table shows 3 local maxima more than 8.0mm apart

Height threshold: T = 4.30, p = 0.001 (1.000)
Extent threshold: k = 0 voxels
Expected voxels per cluster, <k> = 1.411
Expected number of clusters, <c> = 45.53
FWEp: 11.012, FDRp: Inf, FWEc: Inf, FDRc: Inf

Degrees of freedom = [1.0, 9.0]
FWHM = 9.5 10.0 8.3 mm mm mm; 3.2 3.3 2.8 {voxels}
Volume: 1692981 = 62703 voxels = 1935.4 resels
Voxel size: 3.0 3.0 3.0 mm mm mm; (resel = 29.33 voxels)
