# Supplementary material for: Arterial spin labeling versus BOLD in direct challenge and drug-task interaction pharmacological fMRI
Source: PeerJ. 2014 Dec 11;2:e687. doi: 10.7717/peerj.687 (PMC4266850; doi:10.7717/peerj.687)
Supplement: Figure S2 — First three pages show activation clusters and last three pages show deactivation clusters. [file peerj-02-687-s008.pdf]

A BOLD 2 back effect increases 60 mg only

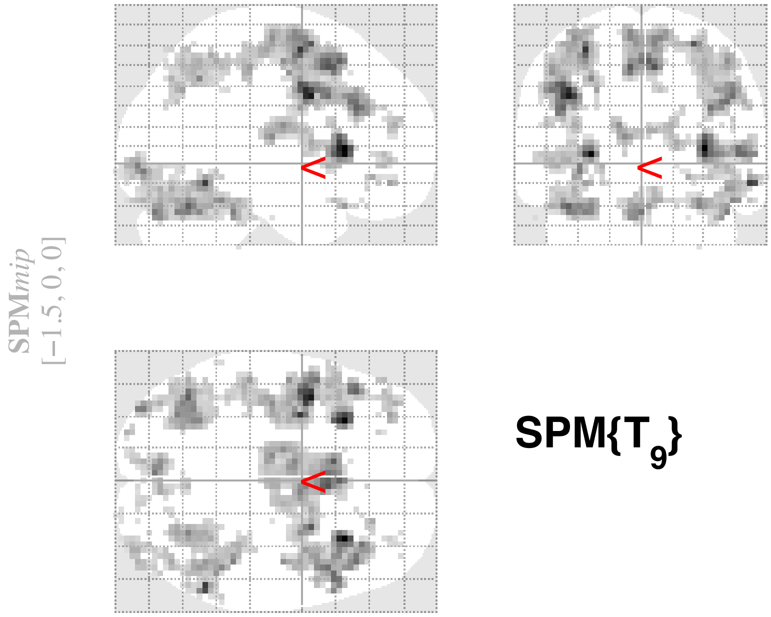

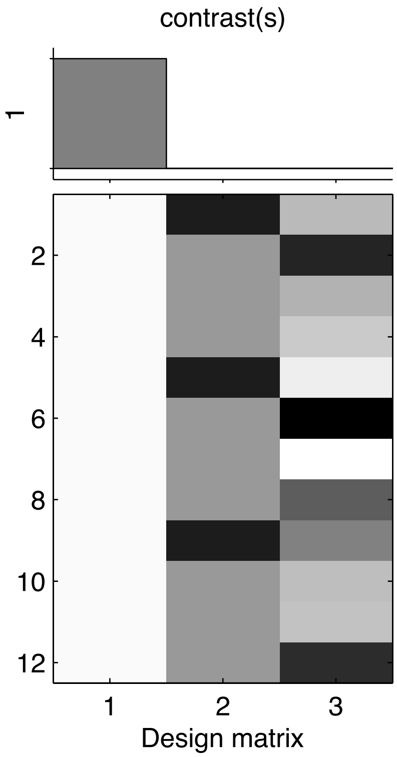

SPMresults: ./bold_2back_effect_60_only
Height threshold T = 4.296806 {p<0.001 (unc.)}
Extent threshold k = 0 voxels

Statistics: ***p−values adjusted for search volume***

set−level		cluster−level				peak−level					mm mm mm		
p	c	$p_{FWE-corr}$	$q_{FDR-corr}$	k_E	p_{uncorr}	$p_{FWE-corr}$	$q_{FDR-corr}$	T	$(Z_{\equiv})$	p_{uncorr}			
**0.001**	**48**	**0.000**	**0.000**	**142**	**0.000**	**0.009**	**0.310**	**13.42**	**5.13**	**0.000**	**32**	**21**	**6**
						0.288	0.404	8.92	4.44	0.000	50	15	3
		**0.000**	**0.000**	**127**	**0.000**	**0.013**	**0.310**	**12.94**	**5.07**	**0.000**	**−28**	**21**	**3**
						0.998	0.522	6.29	3.80	0.000	−46	12	−3
						0.999	0.522	6.23	3.78	0.000	−40	18	0
		**0.000**	**0.000**	**515**	**0.000**	**0.020**	**0.310**	**12.29**	**4.98**	**0.000**	**−40**	**3**	**33**
						0.056	0.389	10.87	4.78	0.000	−32	−3	57
						0.245	0.404	9.10	4.47	0.000	−44	27	27
		**0.000**	**0.000**	**327**	**0.000**	**0.061**	**0.389**	**10.75**	**4.76**	**0.000**	**56**	**−54**	**−12**
						0.681	0.404	8.02	4.25	0.000	44	−39	−27
						0.717	0.404	7.97	4.23	0.000	28	−63	−24
		**0.000**	**0.000**	**471**	**0.000**	**0.132**	**0.404**	**9.80**	**4.60**	**0.000**	**4**	**12**	**48**
						0.160	0.404	9.58	4.56	0.000	26	3	60
						0.166	0.404	9.54	4.55	0.000	−8	15	48
		**0.000**	**0.000**	**224**	**0.000**	**0.187**	**0.404**	**9.40**	**4.53**	**0.000**	**−40**	**−63**	**−24**
						0.544	0.404	8.25	4.30	0.000	−34	−87	−6
						0.643	0.404	8.08	4.26	0.000	−32	−69	−27
		**0.000**	**0.000**	**223**	**0.000**	**0.344**	**0.404**	**8.73**	**4.40**	**0.000**	**44**	**27**	**30**
						0.379	0.404	8.62	4.38	0.000	46	12	33
						0.424	0.404	8.51	4.35	0.000	34	30	27
		**0.000**	**0.000**	**108**	**0.000**	**0.466**	**0.404**	**8.41**	**4.33**	**0.000**	**−2**	**−81**	**−27**
						0.996	0.522	6.47	3.86	0.000	8	−78	−21
						0.999	0.524	6.09	3.74	0.000	−8	−87	−21
		**0.003**	**0.000**	**47**	**0.000**	**0.909**	**0.404**	**7.69**	**4.17**	**0.000**	**−28**	**−57**	**42**

table shows 3 local maxima more than 8.0mm apart

Height threshold: T = 4.30, p = 0.001 (1.000)
Extent threshold: k = 0 voxels
Expected voxels per cluster, <k> = 2.250
Expected number of clusters, <c> = 29.13
FWEp: 11.012, FDRp: Inf, FWEc: 47, FDRc: 22

Degrees of freedom = [1.0, 9.0]
FWHM = 10.9 11.3 10.3 mm mm mm; 3.6 3.8 3.4 {voxels}
Volume: 1692981 = 62703 voxels = 1213.8 resels
Voxel size: 3.0 3.0 3.0 mm mm mm; (resel = 46.77 voxels)

BOLD 2 back effect increases 60 mg only

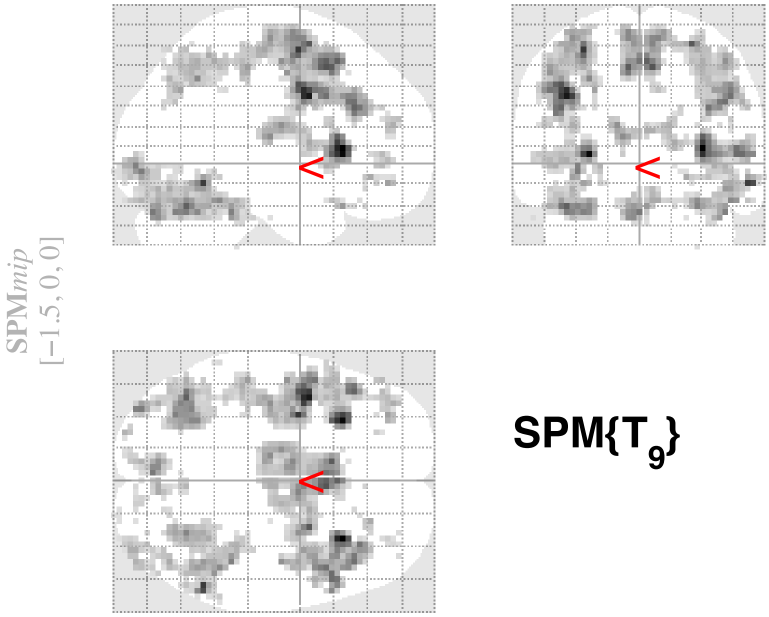

SPM{T_9}

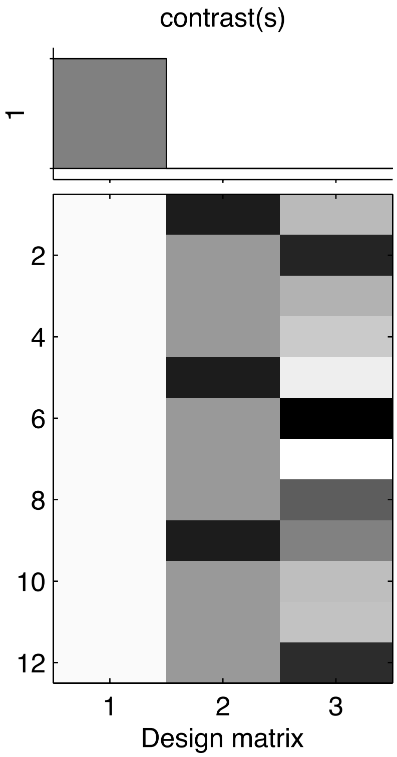

SPMresults: ./bold_2back_effect_60_only
Height threshold T = 4.296806 {p<0.001 (unc.)}
Extent threshold k = 0 voxels

Statistics: ***p-values adjusted for search volume***

set-level		cluster-level				peak-level					mm mm mm		
p	c	$p_{FWE-corr}$	$q_{FDR-corr}$	k_E	p_{uncorr}	$p_{FWE-corr}$	$q_{FDR-corr}$	T	$(Z_{\equiv})$	p_{uncorr}			
						0.990	0.522	6.72	3.93	0.000	-26	-66	36
						0.999	0.522	6.14	3.76	0.000	-28	-72	42
		**0.000**	**0.000**	**166**	**0.000**	**0.932**	**0.432**	**7.53**	**4.13**	**0.000**	**-10**	**-18**	**12**
						0.952	0.436	7.34	4.09	0.000	-14	-12	18
						0.996	0.522	6.44	3.85	0.000	16	3	9
		**0.517**	**0.086**	**12**	**0.025**	**0.971**	**0.487**	**7.11**	**4.03**	**0.000**	**2**	**21**	**-24**
		**0.942**	**0.247**	**6**	**0.098**	**0.989**	**0.522**	**6.77**	**3.94**	**0.000**	**-32**	**45**	**-12**
		**0.942**	**0.247**	**6**	**0.098**	**0.997**	**0.522**	**6.40**	**3.83**	**0.000**	**22**	**36**	**-9**
		**0.000**	**0.000**	**163**	**0.000**	**0.997**	**0.522**	**6.38**	**3.83**	**0.000**	**44**	**-48**	**51**
						0.998	0.522	6.28	3.80	0.000	32	-57	51
						1.000	0.586	5.86	3.67	0.000	32	-66	48
		**0.109**	**0.016**	**22**	**0.004**	**0.998**	**0.522**	**6.30**	**3.81**	**0.000**	**-38**	**48**	**18**
						1.000	0.728	5.10	3.41	0.000	-34	42	27
		**0.891**	**0.228**	**7**	**0.076**	**0.999**	**0.522**	**6.15**	**3.76**	**0.000**	**-22**	**-96**	**-3**
		**0.386**	**0.062**	**14**	**0.017**	**1.000**	**0.605**	**5.80**	**3.65**	**0.000**	**-44**	**42**	**3**
		**0.999**	**0.370**	**3**	**0.231**	**1.000**	**0.612**	**5.76**	**3.64**	**0.000**	**-50**	**42**	**-3**
		**0.750**	**0.152**	**9**	**0.048**	**1.000**	**0.643**	**5.49**	**3.55**	**0.000**	**-52**	**15**	**18**
		**0.976**	**0.292**	**5**	**0.128**	**1.000**	**0.703**	**5.27**	**3.47**	**0.000**	**-44**	**-51**	**48**
		**0.999**	**0.370**	**3**	**0.231**	**1.000**	**0.707**	**5.24**	**3.46**	**0.000**	**46**	**15**	**21**
		**0.993**	**0.339**	**4**	**0.170**	**1.000**	**0.728**	**5.17**	**3.44**	**0.000**	**-10**	**0**	**3**
		**0.999**	**0.370**	**3**	**0.231**	**1.000**	**0.728**	**5.09**	**3.41**	**0.000**	**-50**	**-54**	**-15**
		**0.999**	**0.370**	**3**	**0.231**	**1.000**	**0.728**	**5.06**	**3.40**	**0.000**	**-22**	**39**	**-12**
		**0.942**	**0.247**	**6**	**0.098**	**1.000**	**0.731**	**5.04**	**3.39**	**0.000**	**32**	**45**	**21**
		**1.000**	**0.495**	**1**	**0.495**	**1.000**	**0.813**	**4.86**	**3.32**	**0.000**	**-44**	**33**	**-15**

table shows 3 local maxima more than 8.0mm apart

Height threshold: T = 4.30, p = 0.001 (1.000)
Extent threshold: k = 0 voxels
Expected voxels per cluster, <k> = 2.250
Expected number of clusters, <c> = 29.13
FWEp: 11.012, FDRp: Inf, FWEc: 47, FDRc: 22

Degrees of freedom = [1.0, 9.0]
FWHM = 10.9 11.3 10.3 mm mm mm; 3.6 3.8 3.4 {voxels}
Volume: 1692981 = 62703 voxels = 1213.8 resels
Voxel size: 3.0 3.0 3.0 mm mm mm; (resel = 46.77 voxels)

BOLD 2 back effect increases 60 mg only

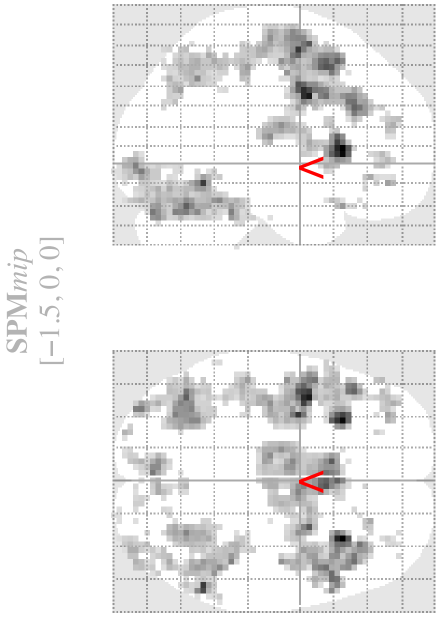

SPM{T_9}

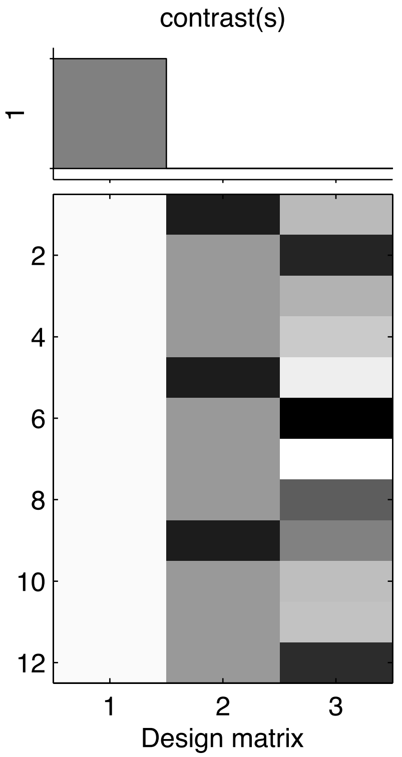

SPMresults: /bold_2back_effect_60_only
Height threshold T = 4.296806 {p<0.001 (unc.)}
Extent threshold k = 0 voxels

Statistics: *p−values adjusted for search volume*

set−level		cluster−level				peak−level					mm mm mm		
p	c	$p_{FWE-corr}$	$q_{FDR-corr}$	k_E	p_{uncorr}	$p_{FWE-corr}$	$q_{FDR-corr}$	T	$(Z_{\equiv})$	p_{uncorr}			
		0.999	0.370	3	0.231	1.000	0.813	4.85	3.32	0.000	20	−72	57
		1.000	0.495	1	0.495	1.000	0.827	4.82	3.30	0.000	28	−84	−18
		1.000	0.495	1	0.495	1.000	0.837	4.79	3.29	0.000	4	33	−24
		0.999	0.370	3	0.231	1.000	0.837	4.77	3.28	0.001	−34	−30	−27
		1.000	0.476	2	0.327	1.000	0.849	4.73	3.27	0.001	−26	−75	27
		0.993	0.339	4	0.170	1.000	0.854	4.71	3.26	0.001	8	−72	48
		1.000	0.476	2	0.327	1.000	0.858	4.69	3.25	0.001	−40	−66	−3
		1.000	0.495	1	0.495	1.000	0.862	4.67	3.25	0.001	16	27	−24
		0.976	0.292	5	0.128	1.000	0.877	4.64	3.24	0.001	−40	−48	39
		1.000	0.495	1	0.495	1.000	0.891	4.59	3.21	0.001	−14	21	−24
		1.000	0.476	2	0.327	1.000	0.905	4.55	3.20	0.001	−58	−45	−30
		1.000	0.495	1	0.495	1.000	0.914	4.53	3.19	0.001	20	30	−21
		1.000	0.495	1	0.495	1.000	0.915	4.51	3.18	0.001	40	51	18
		1.000	0.495	1	0.495	1.000	0.918	4.49	3.17	0.001	28	51	18
		0.993	0.339	4	0.170	1.000	0.937	4.44	3.15	0.001	20	−99	−6
		1.000	0.495	1	0.495	1.000	0.937	4.44	3.15	0.001	−8	24	45
		1.000	0.495	1	0.495	1.000	0.949	4.41	3.14	0.001	−34	−60	60
		1.000	0.495	1	0.495	1.000	0.949	4.40	3.14	0.001	−20	−96	−12
		1.000	0.495	1	0.495	1.000	0.962	4.37	3.12	0.001	22	−90	−21
		1.000	0.495	1	0.495	1.000	0.971	4.35	3.11	0.001	−32	48	12
		1.000	0.495	1	0.495	1.000	0.978	4.33	3.10	0.001	22	−18	−30
		1.000	0.495	1	0.495	1.000	0.978	4.33	3.10	0.001	28	−36	−45

table shows 3 local maxima more than 8.0mm apart

Height threshold: T = 4.30, p = 0.001 (1.000)
Extent threshold: k = 0 voxels
Expected voxels per cluster, <k> = 2.250
Expected number of clusters, <c> = 29.13
FWEp: 11.012, FDRp: Inf, FWEc: 47, FDRc: 22

Degrees of freedom = [1.0, 9.0]
FWHM = 10.9 11.3 10.3 mm mm mm; 3.6 3.8 3.4 {voxels}
Volume: 1692981 = 62703 voxels = 1213.8 resels
Voxel size: 3.0 3.0 3.0 mm mm mm; (resel = 46.77 voxels)

B

BOLD 2 back effect decreases 60 mg

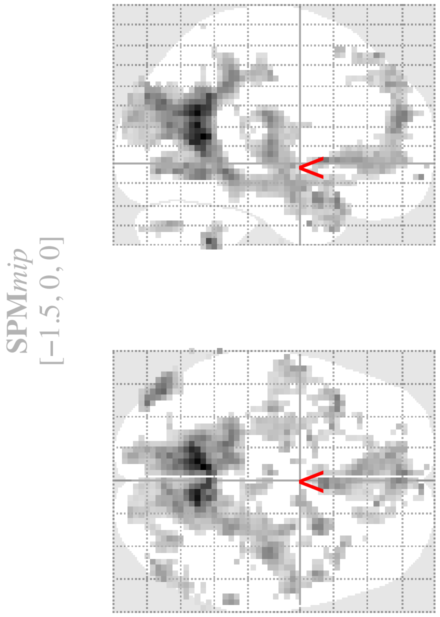

SPM{T_9}

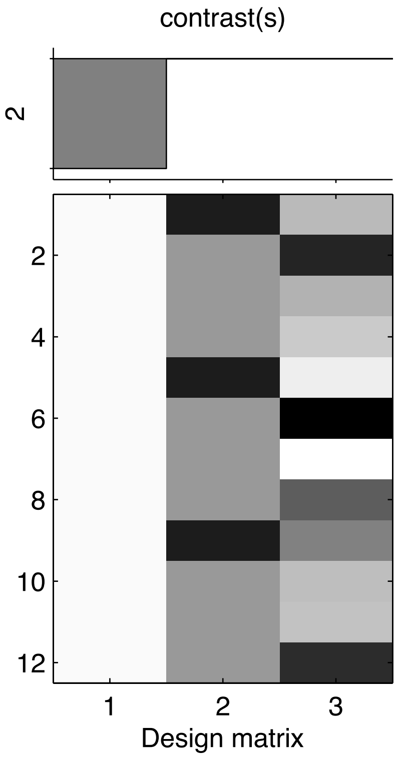

SPMresults: /bold_2back_effect_60_only
Height threshold T = 4.296806 {p<0.001 (unc.)}
Extent threshold k = 0 voxels

Statistics: *p-values adjusted for search volume*

set-level		cluster-level				peak-level					mm mm mm
p	c	$p_{FWE-corr}$	$q_{FDR-corr}$	k_E	p_{uncorr}	$p_{FWE-corr}$	$q_{FDR-corr}$	T	$(Z_{\equiv})$	p_{uncorr}	
**0.000**	**52**	**0.000**	**0.000**	**2142**	**0.000**	**0.015**	**0.441**	**12.70**	**5.04**	**0.000**	**-4 -54 12**
						0.020	0.441	12.25	4.98	0.000	-8 -57 24
						0.025	0.441	11.96	4.94	0.000	10 -57 18
		**0.003**	**0.001**	**46**	**0.000**	**0.140**	**0.541**	**9.74**	**4.59**	**0.000**	**10 -51 -42**
						1.000	0.595	5.88	3.68	0.000	14 -45 -36
		**0.000**	**0.000**	**132**	**0.000**	**0.327**	**0.541**	**8.78**	**4.41**	**0.000**	**-44 -75 30**
						0.525	0.541	8.28	4.30	0.000	-38 -84 33
						1.000	0.740	5.13	3.42	0.000	-44 -87 12
		**0.000**	**0.000**	**507**	**0.000**	**0.671**	**0.541**	**8.03**	**4.25**	**0.000**	**4 12 0**
						0.776	0.541	7.89	4.22	0.000	-2 54 24
						0.980	0.541	6.97	3.99	0.000	-10 45 39
		**0.000**	**0.000**	**360**	**0.000**	**0.883**	**0.541**	**7.76**	**4.19**	**0.000**	**38 -18 21**
						0.911	0.541	7.69	4.17	0.000	38 -18 3
						0.972	0.541	7.10	4.03	0.000	50 6 -21
		**0.001**	**0.000**	**59**	**0.000**	**0.926**	**0.541**	**7.57**	**4.14**	**0.000**	**26 6 -21**
						0.999	0.585	6.24	3.79	0.000	14 0 -12
						0.999	0.585	6.16	3.76	0.000	22 12 -12
		**0.037**	**0.006**	**29**	**0.001**	**0.966**	**0.541**	**7.18**	**4.05**	**0.000**	**14 39 54**
						1.000	0.638	5.66	3.61	0.000	20 30 54
		**0.891**	**0.264**	**7**	**0.076**	**0.968**	**0.541**	**7.15**	**4.04**	**0.000**	**4 63 -9**
		**0.825**	**0.222**	**8**	**0.060**	**0.974**	**0.541**	**7.06**	**4.02**	**0.000**	**-20 3 -33**
						1.000	0.833	4.83	3.31	0.000	-28 6 -36
		**0.750**	**0.190**	**9**	**0.048**	**0.983**	**0.541**	**6.91**	**3.98**	**0.000**	**64 -39 42**
		**0.000**	**0.000**	**65**	**0.000**	**0.987**	**0.541**	**6.81**	**3.95**	**0.000**	**-56 0 -15**

table shows 3 local maxima more than 8.0mm apart

Height threshold: T = 4.30, p = 0.001 (1.000)
Extent threshold: k = 0 voxels
Expected voxels per cluster, <k> = 2.250
Expected number of clusters, <c> = 29.13
FWEp: 11.012, FDRp: Inf, FWEc: 29, FDRc: 29

Degrees of freedom = [1.0, 9.0]
FWHM = 10.9 11.3 10.3 mm mm mm; 3.6 3.8 3.4 {voxels}
Volume: 1692981 = 62703 voxels = 1213.8 resels
Voxel size: 3.0 3.0 3.0 mm mm mm; (resel = 46.77 voxels)

BOLD 2 back effect decreases 60 mg

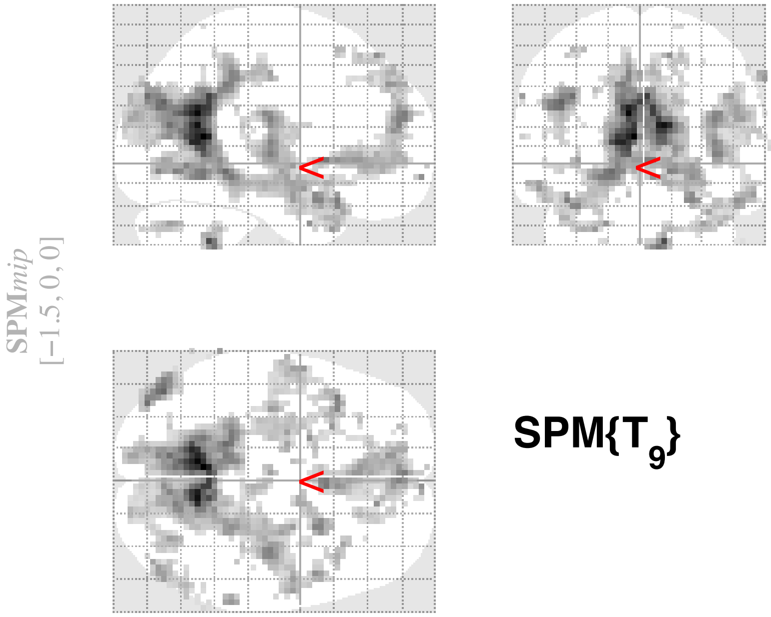

SPM{T_9}

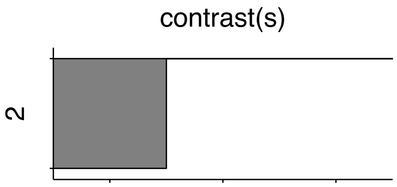

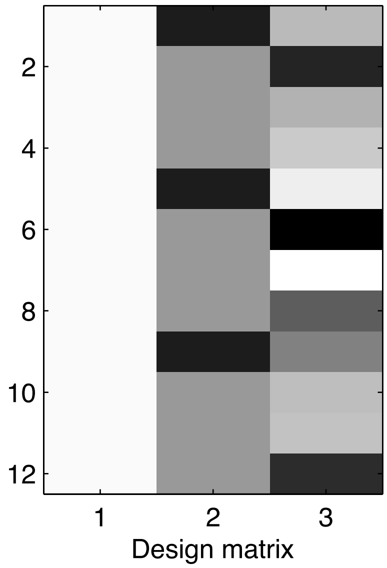

SPMresults: /bold_2back_effect_60_only
Height threshold T = 4.296806 {p<0.001 (unc.)}
Extent threshold k = 0 voxels

Statistics: *p–values adjusted for search volume*

set–level		cluster–level				peak–level					mm mm mm
p	c	$p_{FWE-corr}$	$q_{FDR-corr}$	k_E	p_{uncorr}	$p_{FWE-corr}$	$q_{FDR-corr}$	T	$(Z_{\equiv})$	p_{uncorr}	
						0.999	0.585	6.03	3.73	0.000	-62 -9 -12
						1.000	0.833	4.81	3.30	0.000	-50 6 -18
		**0.000**	**0.000**	**104**	**0.000**	**0.990**	**0.541**	**6.72**	**3.93**	**0.000**	**52 -75 21**
						0.991	0.541	6.70	3.92	0.000	46 -78 33
						1.000	0.606	5.84	3.67	0.000	56 -63 24
		**1.000**	**0.486**	**2**	**0.327**	**0.990**	**0.541**	**6.72**	**3.92**	**0.000**	**-64 -45 33**
		**0.008**	**0.001**	**40**	**0.000**	**0.991**	**0.541**	**6.68**	**3.91**	**0.000**	**-34 -18 0**
						1.000	0.761	5.04	3.39	0.000	-44 -15 -6
		**0.006**	**0.001**	**42**	**0.000**	**0.995**	**0.541**	**6.50**	**3.87**	**0.000**	**32 -72 -33**
						1.000	0.788	4.98	3.37	0.000	20 -87 -36
		**0.942**	**0.282**	**6**	**0.098**	**1.000**	**0.607**	**5.81**	**3.65**	**0.000**	**-38 18 57**
		**0.993**	**0.367**	**4**	**0.170**	**1.000**	**0.672**	**5.55**	**3.57**	**0.000**	**-8 -6 -18**
		**0.942**	**0.282**	**6**	**0.098**	**1.000**	**0.672**	**5.52**	**3.56**	**0.000**	**-28 30 -6**
		**0.993**	**0.367**	**4**	**0.170**	**1.000**	**0.675**	**5.51**	**3.56**	**0.000**	**-26 51 36**
		**0.448**	**0.088**	**13**	**0.020**	**1.000**	**0.692**	**5.45**	**3.53**	**0.000**	**-16 6 -12**
		**1.000**	**0.486**	**2**	**0.327**	**1.000**	**0.721**	**5.31**	**3.49**	**0.000**	**44 -36 27**
		**0.993**	**0.367**	**4**	**0.170**	**1.000**	**0.732**	**5.24**	**3.46**	**0.000**	**-26 -72 -33**
		**1.000**	**0.495**	**1**	**0.495**	**1.000**	**0.732**	**5.22**	**3.46**	**0.000**	**-4 66 -3**
		**0.942**	**0.282**	**6**	**0.098**	**1.000**	**0.740**	**5.15**	**3.43**	**0.000**	**16 48 45**
		**0.993**	**0.367**	**4**	**0.170**	**1.000**	**0.740**	**5.14**	**3.43**	**0.000**	**16 -24 3**
		**0.999**	**0.429**	**3**	**0.231**	**1.000**	**0.755**	**5.08**	**3.41**	**0.000**	**-2 24 -12**
		**1.000**	**0.495**	**1**	**0.495**	**1.000**	**0.820**	**4.91**	**3.34**	**0.000**	**-64 -60 12**
		**0.976**	**0.349**	**5**	**0.128**	**1.000**	**0.820**	**4.90**	**3.34**	**0.000**	**34 3 18**
		**1.000**	**0.486**	**2**	**0.327**	**1.000**	**0.823**	**4.87**	**3.33**	**0.000**	**4 54 39**

table shows 3 local maxima more than 8.0mm apart

Height threshold: T = 4.30, p = 0.001 (1.000)
Extent threshold: k = 0 voxels
Expected voxels per cluster, <k> = 2.250
Expected number of clusters, <c> = 29.13
FWEp: 11.012, FDRp: Inf, FWEc: 29, FDRc: 29

Degrees of freedom = [1.0, 9.0]
FWHM = 10.9 11.3 10.3 mm mm mm; 3.6 3.8 3.4 {voxels}
Volume: 1692981 = 62703 voxels = 1213.8 resels
Voxel size: 3.0 3.0 3.0 mm mm mm; (resel = 46.77 voxels)

BOLD 2 back effect decreases 60 mg

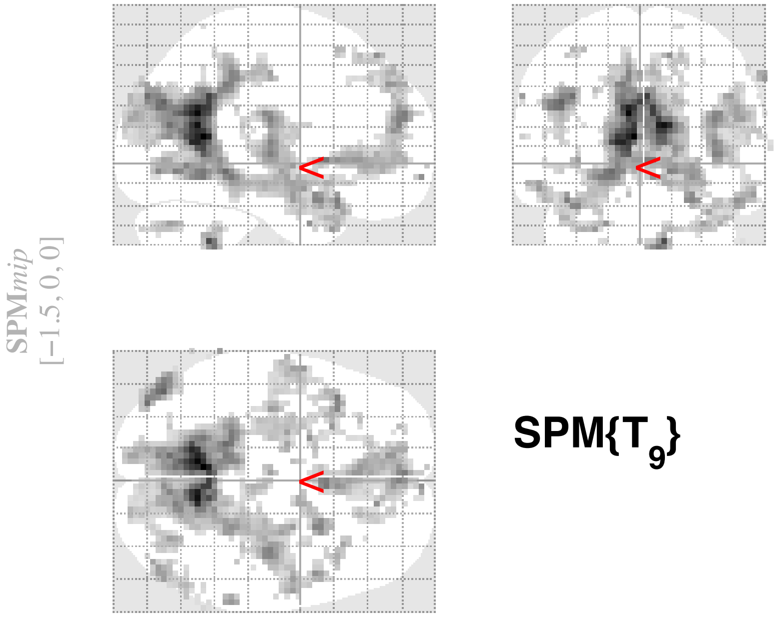

SPM{T_9}

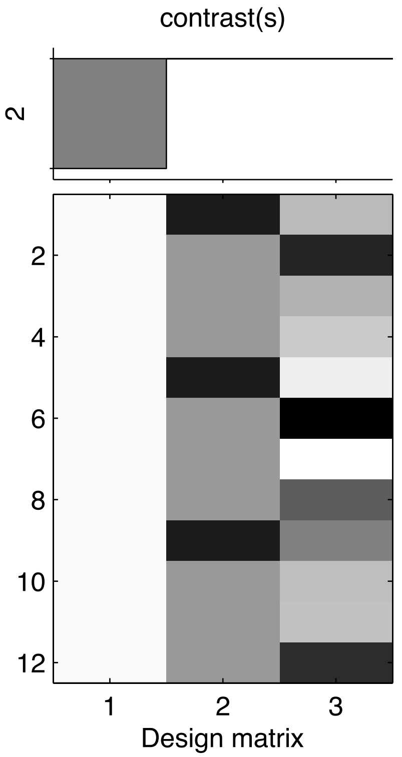

SPMresults: /bold_2back_effect_60_only
Height threshold T = 4.296806 {p<0.001 (unc.)}
Extent threshold k = 0 voxels

Statistics: *p-values adjusted for search volume*

set-level		cluster-level				peak-level					mm mm mm		
p	c	$p_{FWE-corr}$	$q_{FDR-corr}$	k_E	p_{uncorr}	$p_{FWE-corr}$	$q_{FDR-corr}$	T	$(Z_{\equiv})$	p_{uncorr}			
		0.999	0.429	3	0.231	1.000	0.823	4.87	3.33	0.000	-52	-9	12
		0.993	0.367	4	0.170	1.000	0.844	4.75	3.28	0.001	-22	21	-12
		1.000	0.495	1	0.495	1.000	0.862	4.72	3.26	0.001	-14	-9	-21
		0.999	0.429	3	0.231	1.000	0.882	4.66	3.24	0.001	68	-54	6
		1.000	0.495	1	0.495	1.000	0.887	4.64	3.24	0.001	-38	-9	-27
		0.999	0.429	3	0.231	1.000	0.900	4.62	3.22	0.001	56	-54	48
		1.000	0.486	2	0.327	1.000	0.901	4.61	3.22	0.001	8	63	24
		1.000	0.486	2	0.327	1.000	0.906	4.59	3.21	0.001	-20	-48	-42
		1.000	0.495	1	0.495	1.000	0.906	4.58	3.21	0.001	62	-15	42
		1.000	0.495	1	0.495	1.000	0.915	4.55	3.20	0.001	14	54	42
		1.000	0.486	2	0.327	1.000	0.916	4.54	3.19	0.001	20	-54	69
		1.000	0.486	2	0.327	1.000	0.920	4.52	3.19	0.001	-34	-27	21
		1.000	0.495	1	0.495	1.000	0.932	4.49	3.17	0.001	28	9	-30
		1.000	0.495	1	0.495	1.000	0.960	4.43	3.15	0.001	-32	-30	18
		1.000	0.495	1	0.495	1.000	0.960	4.42	3.14	0.001	-62	-51	12
		1.000	0.495	1	0.495	1.000	0.968	4.40	3.13	0.001	-22	48	42
		1.000	0.495	1	0.495	1.000	0.968	4.38	3.13	0.001	-52	-66	15
		1.000	0.495	1	0.495	1.000	0.968	4.38	3.12	0.001	46	-27	15
		1.000	0.495	1	0.495	1.000	0.968	4.36	3.12	0.001	32	33	51
		1.000	0.495	1	0.495	1.000	0.968	4.36	3.12	0.001	52	-12	-42
		1.000	0.495	1	0.495	1.000	0.968	4.35	3.11	0.001	64	-57	24
		1.000	0.495	1	0.495	1.000	0.972	4.34	3.11	0.001	62	-57	33
		1.000	0.495	1	0.495	1.000	0.996	4.30	3.09	0.001	-22	9	-27

table shows 3 local maxima more than 8.0mm apart

Height threshold: T = 4.30, p = 0.001 (1.000)
Extent threshold: k = 0 voxels
Expected voxels per cluster, <k> = 2.250
Expected number of clusters, <c> = 29.13
FWEp: 11.012, FDRp: Inf, FWEc: 29, FDRc: 29

Degrees of freedom = [1.0, 9.0]
FWHM = 10.9 11.3 10.3 mm mm mm; 3.6 3.8 3.4 {voxels}
Volume: 1692981 = 62703 voxels = 1213.8 resels
Voxel size: 3.0 3.0 3.0 mm mm mm; (resel = 46.77 voxels)
