# Supplementary material for: Arterial spin labeling versus BOLD in direct challenge and drug-task interaction pharmacological fMRI
Source: PeerJ. 2014 Dec 11;2:e687. doi: 10.7717/peerj.687 (PMC4266850; doi:10.7717/peerj.687)
Supplement: Figure S7 — First page shows no statistically significant activation clusters and second page shows no statistically significant deactivation clusters. [file peerj-02-687-s013.pdf]

LD increase (pbo and SYN day), 12 subs, 5p7

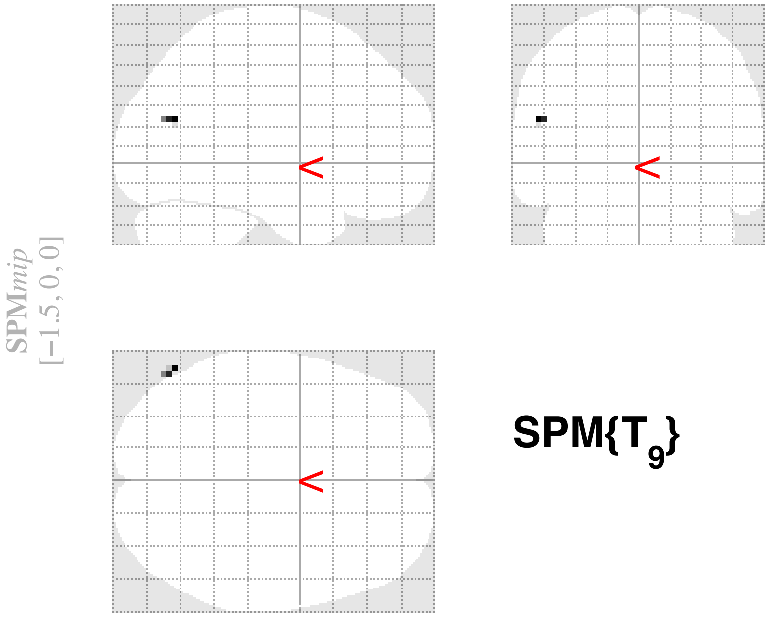

SPM{T_9}

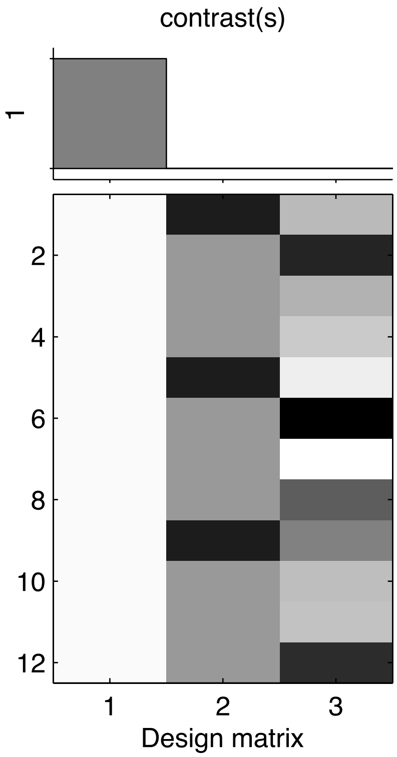

SPMresults: fects_60mg_fixand2back_5p7mm
Height threshold T = 4.296806 {p<0.001 (unc.)}
Extent threshold k = 0 voxels

Statistics: ***p−values adjusted for search volume***

cluster−level				peak−level					mm mm mm
$p_{FWE-corr}$	$q_{FDR-corr}$	k_E	p_{uncorr}	$p_{FWE-corr}$	$q_{FDR-corr}$	T	($Z_{\equiv}$)	p_{uncorr}	
0.905	0.077	5	0.077	1.000	0.538	5.08	3.41	0.000	−56 −69 21

table shows 3 local maxima more than 8.0mm apart

Height threshold: T = 4.30, p = 0.001 (1.000)
Extent threshold: k = 0 voxels
Expected voxels per cluster, <k> = 1.617
Expected number of clusters, <c> = 30.62
FWEp: 10.666, FDRp: Inf, FWEc: Inf, FDRc: Inf

Degrees of freedom = [1.0, 9.0]
FWHM = 9.1 10.4 9.7 mm mm mm; 3.0 3.5 3.2 {voxels}
Volume: 1294110 = 47930 voxels = 1281.4 resels
Voxel size: 3.0 3.0 3.0 mm mm mm; (resel = 33.61 voxels)

LD decreases (Pbo and SYN day), 12 sub

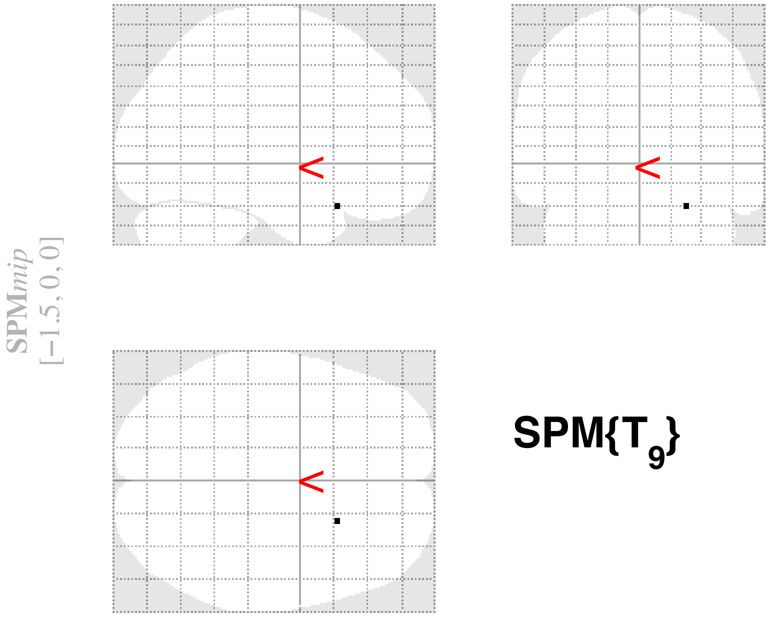

SPM{T_9}

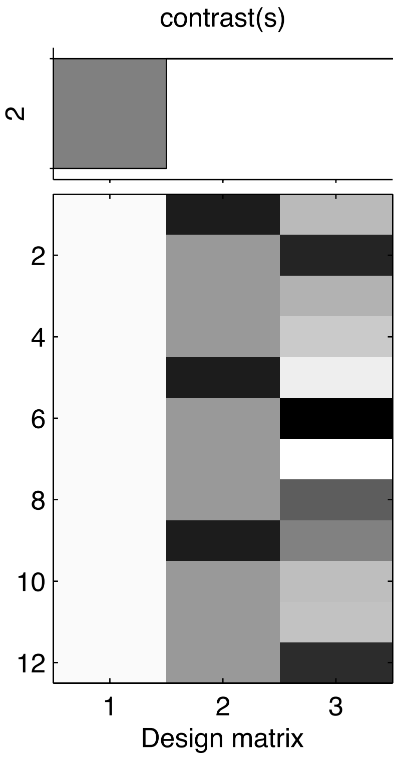

SPMresults:fects_60mg_fixand2back_5p7mm
Height threshold T = 4.296806 {p<0.001 (unc.)}
Extent threshold k = 0 voxels

Statistics: ***p−values adjusted for search volume***

	cluster−level				peak−level					mm mm mm
	$p_{FWE-corr}$	$q_{FDR-corr}$	k_E	p_{uncorr}	$p_{FWE-corr}$	$q_{FDR-corr}$	T	$(Z_{\equiv})$	p_{uncorr}	
	1.000	0.416	1	0.416	1.000	0.541	5.08	3.40	0.000	22 18 −24

table shows 3 local maxima more than 8.0mm apart

Height threshold: T = 4.30, p = 0.001 (1.000)
Extent threshold: k = 0 voxels
Expected voxels per cluster, <k> = 1.617
Expected number of clusters, <c> = 30.62
FWEp: 10.666, FDRp: Inf, FWEc: Inf, FDRc: Inf

Degrees of freedom = [1.0, 9.0]
FWHM = 9.1 10.4 9.7 mm mm mm; 3.0 3.5 3.2 {voxels}
Volume: 1294110 = 47930 voxels = 1281.4 resels
Voxel size: 3.0 3.0 3.0 mm mm mm; (resel = 33.61 voxels)
