# Supplementary material for: Arterial spin labeling versus BOLD in direct challenge and drug-task interaction pharmacological fMRI
Source: PeerJ. 2014 Dec 11;2:e687. doi: 10.7717/peerj.687 (PMC4266850; doi:10.7717/peerj.687)
Supplement: Figure S8 — First page shows no statistically significant activation clusters and second page shows no statistically significant deactivation clusters. [file peerj-02-687-s014.pdf]

BOLD LD increases (pbo and drugd days), 12 subs

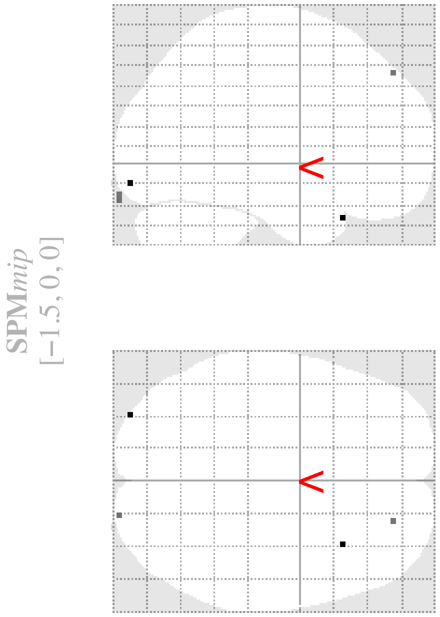

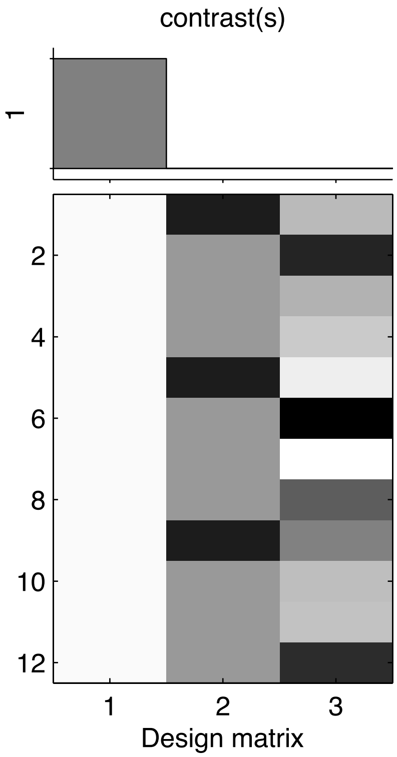

SPM{T_9}

SPMresults: /bold_LD_effect_60_only
Height threshold T = 4.296806 {p<0.001 (unc.)}
Extent threshold k = 0 voxels

Statistics: ***p–values adjusted for search volume***

set–level		cluster–level				peak–level					mm mm mm
p	c	$p_{FWE-corr}$	$q_{FDR-corr}$	k_E	p_{uncorr}	$p_{FWE-corr}$	$q_{FDR-corr}$	T	$(Z_{\equiv})$	p_{uncorr}	
1.000	5	1.000	0.400	1	0.400	1.000	0.883	5.21	3.45	0.000	34 21 −30
		1.000	0.400	1	0.400	1.000	0.883	5.14	3.43	0.000	−32 −93 −12
		1.000	0.400	2	0.233	1.000	0.883	4.74	3.27	0.001	20 −99 −21
		1.000	0.400	1	0.400	1.000	0.883	4.74	3.27	0.001	22 48 45
		1.000	0.400	1	0.400	1.000	0.987	4.31	3.10	0.001	26 −102 −12

table shows 3 local maxima more than 8.0mm apart

Height threshold: T = 4.30, p = 0.001 (1.000)
Extent threshold: k = 0 voxels
Expected voxels per cluster, <k> = 1.514
Expected number of clusters, <c> = 42.57
FWEp: 11.012, FDRp: Inf, FWEc: Inf, FDRc: Inf

Degrees of freedom = [1.0, 9.0]
FWHM = 10.0 10.2 8.3 mm mm mm; 3.3 3.4 2.8 {voxels}
Volume: 1692981 = 62703 voxels = 1804.1 resels
Voxel size: 3.0 3.0 3.0 mm mm mm; (resel = 31.47 voxels)

BOLD LD decreases (pbo and drug days), 12 subs

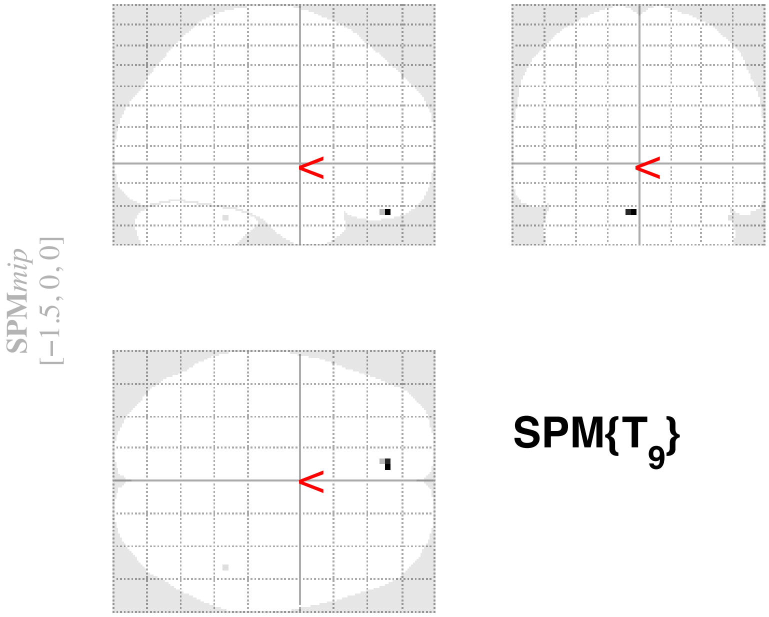

SPM{T_9}

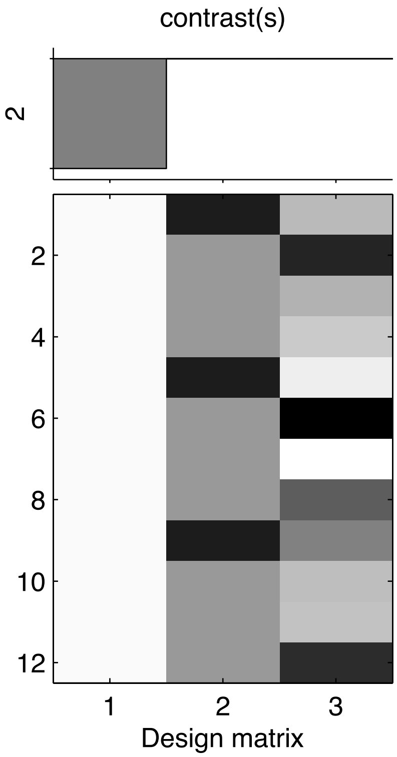

SPMresults: /bold_LD_effect_60_only
Height threshold T = 4.296806 {p<0.001 (unc.)}
Extent threshold k = 0 voxels

Statistics: ***p−values adjusted for search volume***

set−level		cluster−level				peak−level					mm mm mm
p	c	$p_{FWE-corr}$	$q_{FDR-corr}$	k_E	p_{uncorr}	$p_{FWE-corr}$	$q_{FDR-corr}$	T	$(Z_{\equiv})$	p_{uncorr}	
1.000	2	0.998	0.297	3	0.148	0.999	0.331	6.67	3.91	0.000	−4 45 −27
		1.000	0.400	1	0.400	1.000	0.946	4.37	3.12	0.001	46 −42 −30

table shows 3 local maxima more than 8.0mm apart

Height threshold: T = 4.30, p = 0.001 (1.000)
Extent threshold: k = 0 voxels
Expected voxels per cluster, <k> = 1.514
Expected number of clusters, <c> = 42.57
FWEp: 11.012, FDRp: Inf, FWEc: Inf, FDRc: Inf

Degrees of freedom = [1.0, 9.0]
FWHM = 10.0 10.2 8.3 mm mm mm; 3.3 3.4 2.8 {voxels}
Volume: 1692981 = 62703 voxels = 1804.1 resels
Voxel size: 3.0 3.0 3.0 mm mm mm; (resel = 31.47 voxels)
