# Supplementary material for: Arterial spin labeling versus BOLD in direct challenge and drug-task interaction pharmacological fMRI
Source: PeerJ. 2014 Dec 11;2:e687. doi: 10.7717/peerj.687 (PMC4266850; doi:10.7717/peerj.687)
Supplement: Figure S9 — First page shows no statistically significant activation clusters and second page shows no statistically significant deactivation clusters. [file peerj-02-687-s015.pdf]

LD x 2back ASL Increase

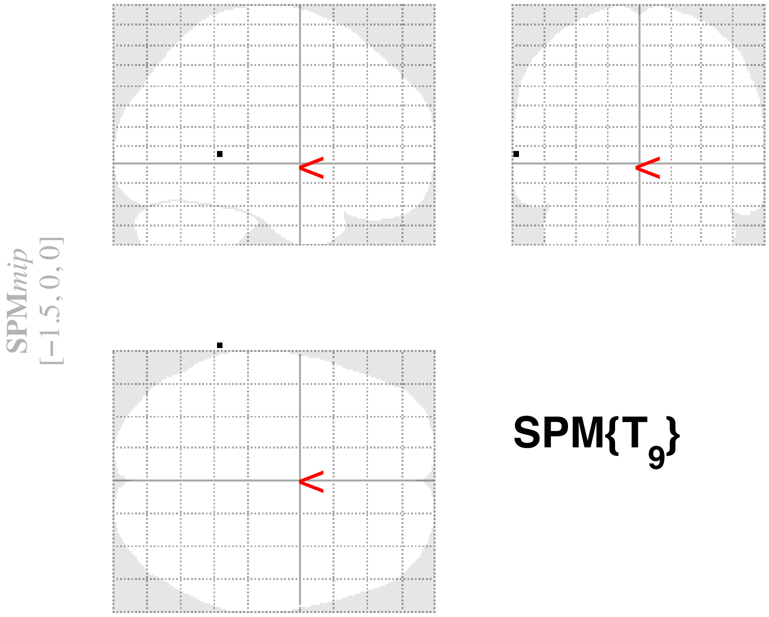

SPM{T_9}

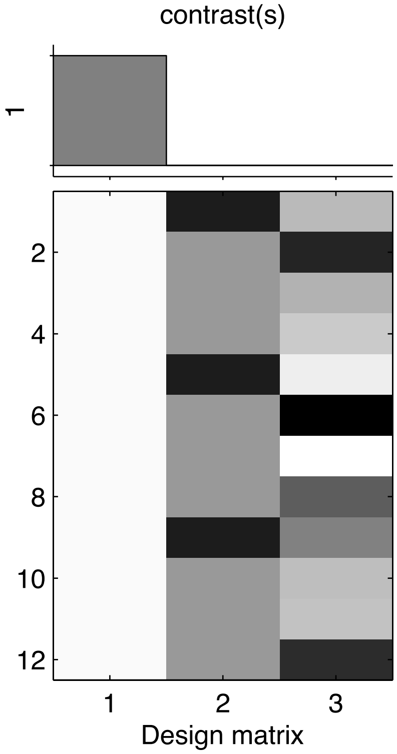

SPMresults: 2back_60mg_fixand2back_5p7mm
Height threshold T = 4.296806 {p<0.001 (unc.)}
Extent threshold k = 0 voxels

Statistics: ***p−values adjusted for search volume***

set-level	cluster-level				peak-level					mm mm mm
	$p_{FWE-corr}$	$q_{FDR-corr}$	k_E	p_{uncorr}	$p_{FWE-corr}$	$q_{FDR-corr}$	T	$(Z_{\equiv})$	p_{uncorr}	
	1.000	0.437	1	0.437	1.000	0.893	4.44	3.15	0.001	−68 −45 3

table shows 3 local maxima more than 8.0mm apart

Height threshold: T = 4.30, p = 0.001 (1.000)
Extent threshold: k = 0 voxels
Expected voxels per cluster, <k> = 1.767
Expected number of clusters, <c> = 28.17
FWEp: 10.666, FDRp: Inf, FWEc: Inf, FDRc: Inf

Degrees of freedom = [1.0, 9.0]
FWHM = 9.0 10.2 10.7 mm mm mm; 3.0 3.4 3.6 {voxels}
Volume: 1294110 = 47930 voxels = 1172.2 resels
Voxel size: 3.0 3.0 3.0 mm mm mm; (resel = 36.74 voxels)

LD x 2back ASL decreases

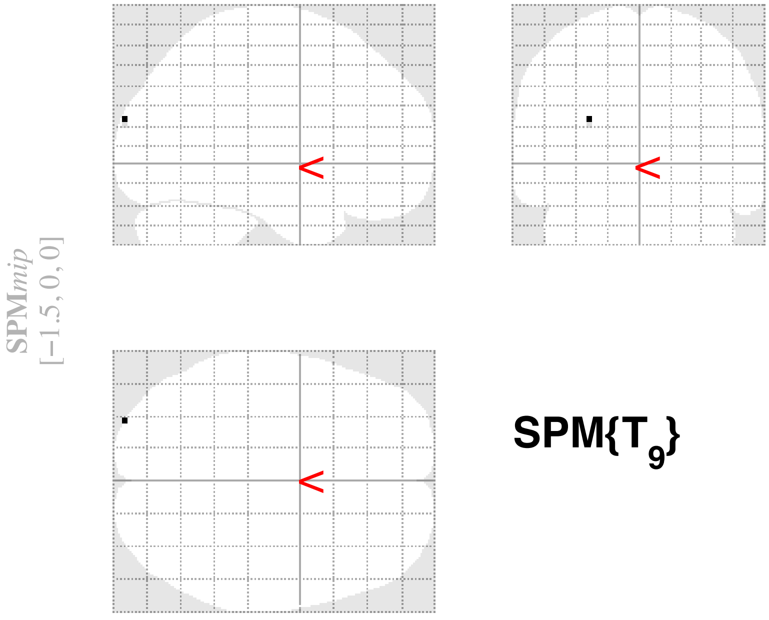

SPM{T_9}

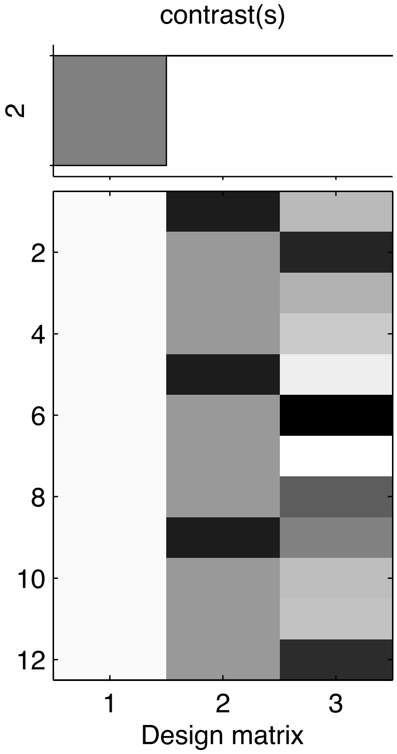

SPMresults: 2back_60mg_fixand2back_5p7mm
Height threshold T = 4.296806 {p<0.001 (unc.)}
Extent threshold k = 0 voxels

Statistics: *p−values adjusted for search volume*

	cluster−level				peak−level					mm mm mm
	$p_{FWE-corr}$	$q_{FDR-corr}$	k_E	p_{uncorr}	$p_{FWE-corr}$	$q_{FDR-corr}$	T	$(Z_{\equiv})$	p_{uncorr}	
	0.999	0.269	2	0.269	1.000	0.629	4.88	3.33	0.000	−28 −96 21

table shows 3 local maxima more than 8.0mm apart

Height threshold: T = 4.30, p = 0.001 (1.000)
Extent threshold: k = 0 voxels
Expected voxels per cluster, <k> = 1.767
Expected number of clusters, <c> = 28.17
FWEp: 10.666, FDRp: Inf, FWEc: Inf, FDRc: Inf

Degrees of freedom = [1.0, 9.0]
FWHM = 9.0 10.2 10.7 mm mm mm; 3.0 3.4 3.6 {voxels}
Volume: 1294110 = 47930 voxels = 1172.2 resels
Voxel size: 3.0 3.0 3.0 mm mm mm; (resel = 36.74 voxels)
