# Supplementary material for: Arterial spin labeling versus BOLD in direct challenge and drug-task interaction pharmacological fMRI
Source: PeerJ. 2014 Dec 11;2:e687. doi: 10.7717/peerj.687 (PMC4266850; doi:10.7717/peerj.687)
Supplement: Figure S10 — First page shows no statistically significant activation clusters and second page shows no statistically significant deactivation clusters. [file peerj-02-687-s016.pdf]

BOLD LDopa on 2back increases 60 mg only

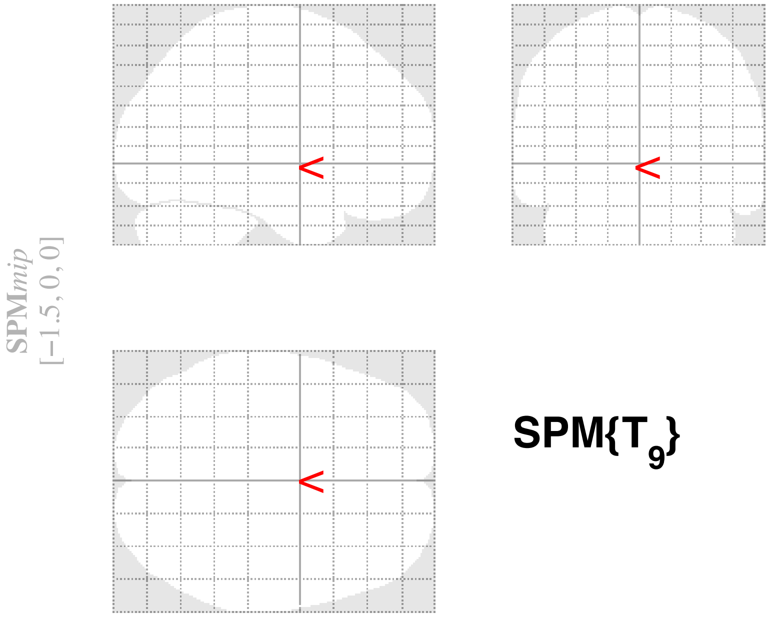

SPM{T_9}

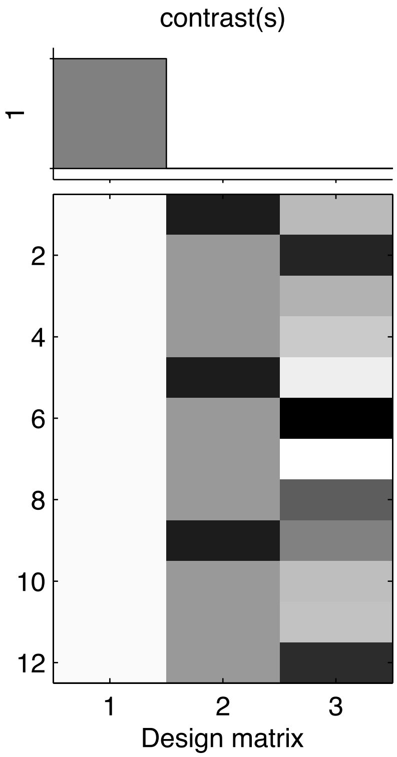

SPMresults: /bold_LDopa_on_2back_60_only
Height threshold T = 4.296806 {p<0.001 (unc.)}
Extent threshold k = 0 voxels

Statistics: ***p−values adjusted for search volume***

set−level		cluster−level				peak−level					mm mm mm
p	*c*	$p_{FWE-corr}$	$q_{FDR-corr}$	k_E	p_{uncorr}	$p_{FWE-corr}$	$q_{FDR-corr}$	*T*	($Z_{\equiv}$)	p_{uncorr}	

no suprathreshold clusters

table shows 3 local maxima more than 8.0mm apart

Height threshold: T = 4.30, p = 0.001 (1.000)
Extent threshold: k = 0 voxels
Expected voxels per cluster, <k> = 1.532
Expected number of clusters, <c> = 42.06
FWEp: 11.012, FDRp: Inf, FWEc: Inf, FDRc: Inf

Degrees of freedom = [1.0, 9.0]
FWHM = 9.7 10.1 8.8 mm mm mm; 3.2 3.4 2.9 {voxels}
Volume: 1692981 = 62703 voxels = 1782.1 resels
Voxel size: 3.0 3.0 3.0 mm mm mm; (resel = 31.85 voxels)

BOLD LDopa on 2back decreases 60 mg only

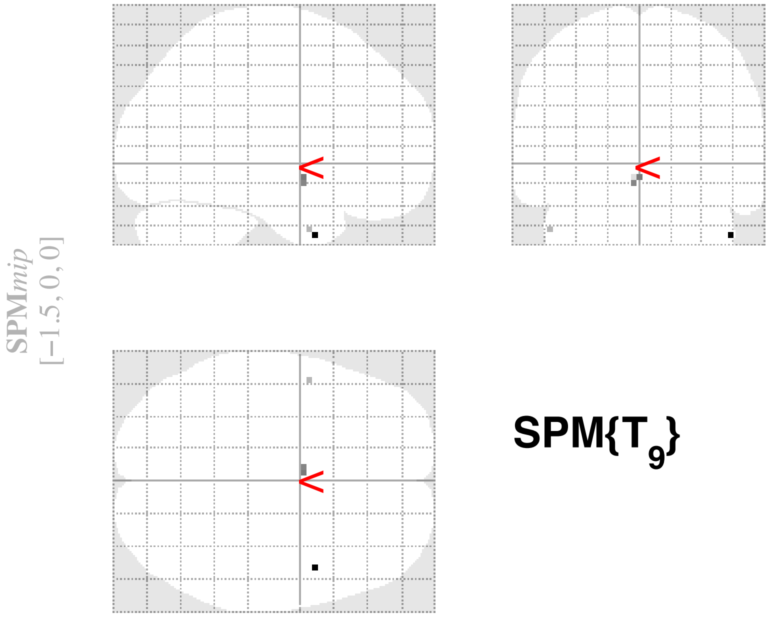

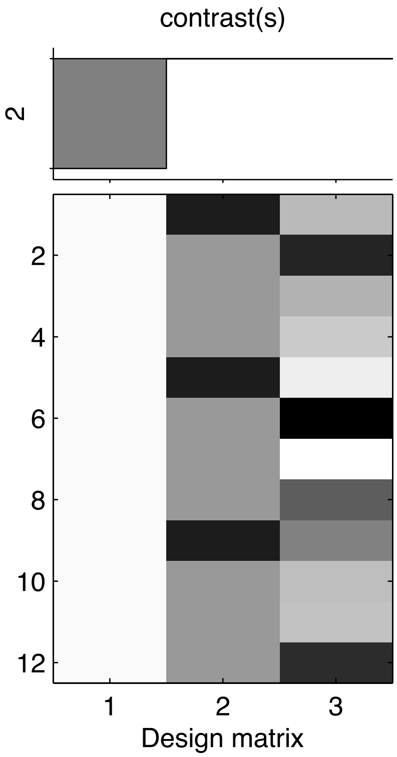

SPMresults: /bold_LDopa_on_2back_60_only
Height threshold T = 4.296806 {p<0.001 (unc.)}
Extent threshold k = 0 voxels

Statistics: *p-values adjusted for search volume*

set-level		cluster-level				peak-level					mm mm mm
p	c	$p_{FWE-corr}$	$q_{FDR-corr}$	k_E	p_{uncorr}	$p_{FWE-corr}$	$q_{FDR-corr}$	T	$(Z_{\equiv})$	p_{uncorr}	
1.000	3	1.000	0.403	1	0.403	1.000	0.824	5.52	3.56	0.000	46 6 −39
		0.998	0.403	3	0.151	1.000	0.824	4.86	3.32	0.000	−2 0 −9
		1.000	0.403	1	0.403	1.000	0.824	4.54	3.19	0.001	−50 3 −36

table shows 3 local maxima more than 8.0mm apart

Height threshold: T = 4.30, p = 0.001 (1.000)
Extent threshold: k = 0 voxels
Expected voxels per cluster, <k> = 1.532
Expected number of clusters, <c> = 42.06
FWEp: 11.012, FDRp: Inf, FWEc: Inf, FDRc: Inf

Degrees of freedom = [1.0, 9.0]
FWHM = 9.7 10.1 8.8 mm mm mm; 3.2 3.4 2.9 {voxels}
Volume: 1692981 = 62703 voxels = 1782.1 resels
Voxel size: 3.0 3.0 3.0 mm mm mm; (resel = 31.85 voxels)
