# Supplementary material for: Arterial spin labeling versus BOLD in direct challenge and drug-task interaction pharmacological fMRI
Source: PeerJ. 2014 Dec 11;2:e687. doi: 10.7717/peerj.687 (PMC4266850; doi:10.7717/peerj.687)
Supplement: Figure S11 — First page shows no statistically significant activation clusters and second page shows no statistically significant deactivation clusters. [file peerj-02-687-s017.pdf]

SYN x 2back x LD increases, 60 mg only, 5p7mm

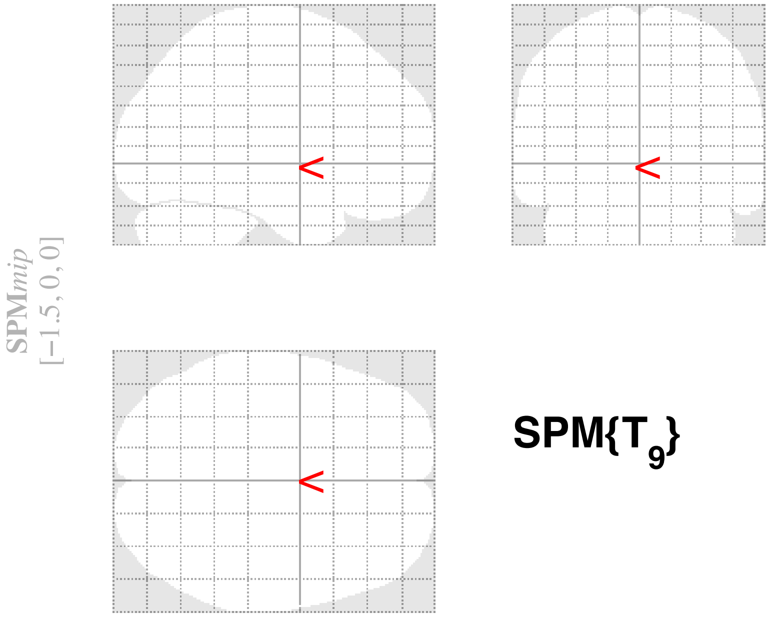

SPM{T_9}

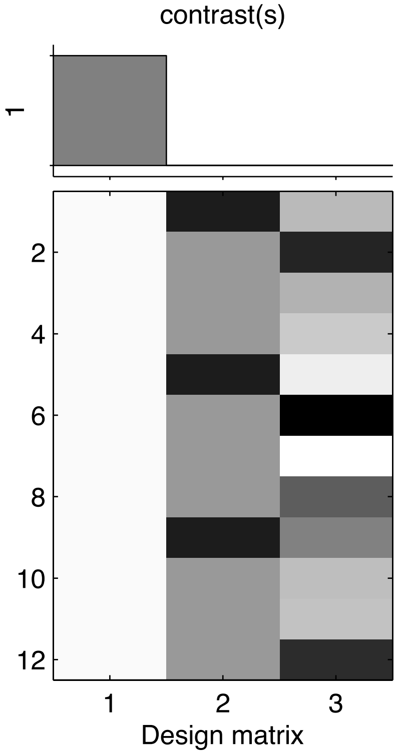

SPMresults: /SYNx2backxLD_60mg_only_5p7mm
Height threshold T = 4.296806 {p<0.001 (unc.)}
Extent threshold k = 0 voxels

Statistics: ***p−values adjusted for search volume***

set−level		cluster−level				peak−level					mm mm mm
p	*c*	$p_{FWE-corr}$	$q_{FDR-corr}$	k_E	p_{uncorr}	$p_{FWE-corr}$	$q_{FDR-corr}$	*T*	($Z_{\equiv}$)	p_{uncorr}	

no suprathreshold clusters

table shows 3 local maxima more than 8.0mm apart

Height threshold: T = 4.30, p = 0.001 (1.000)
Extent threshold: k = 0 voxels
Expected voxels per cluster, <k> = 1.935
Expected number of clusters, <c> = 25.84
FWEp: 10.666, FDRp: Inf, FWEc: Inf, FDRc: Inf

Degrees of freedom = [1.0, 9.0]
FWHM = 9.4 10.5 11.0 mm mm mm; 3.1 3.5 3.7 {voxels}
Volume: 1294110 = 47930 voxels = 1070.5 resels
Voxel size: 3.0 3.0 3.0 mm mm mm; (resel = 40.23 voxels)

SYN x 2back x LD decreases, 60 mg only, 5p7mm

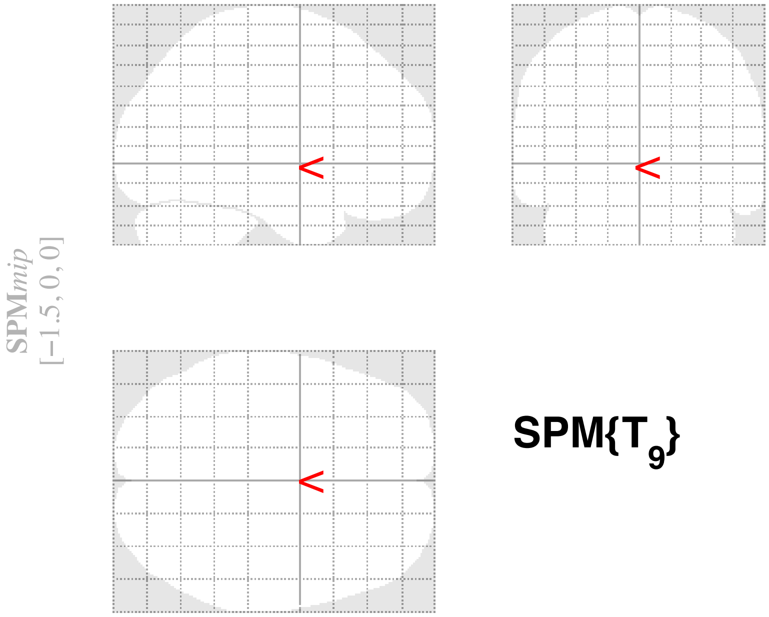

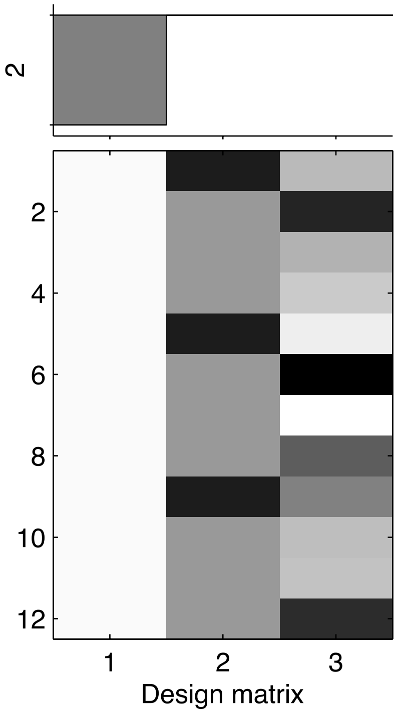

SPM{T_9}

SPMresults: /SYNx2backxLD_60mg_only_5p7mm
Height threshold T = 4.296806 {p<0.001 (unc.)}
Extent threshold k = 0 voxels

Statistics: ***p−values adjusted for search volume***

set−level		cluster−level				peak−level					mm mm mm
p	c	$p_{FWE-corr}$	$q_{FDR-corr}$	k_E	p_{uncorr}	$p_{FWE-corr}$	$q_{FDR-corr}$	T	$(Z_\equiv)$	p_{uncorr}	

no suprathreshold clusters

table shows 3 local maxima more than 8.0mm apart

Height threshold: T = 4.30, p = 0.001 (1.000)
Extent threshold: k = 0 voxels
Expected voxels per cluster, <k> = 1.935
Expected number of clusters, <c> = 25.84
FWEp: 10.666, FDRp: Inf, FWEc: Inf, FDRc: Inf

Degrees of freedom = [1.0, 9.0]
FWHM = 9.4 10.5 11.0 mm mm mm; 3.1 3.5 3.7 {voxels}
Volume: 1294110 = 47930 voxels = 1070.5 resels
Voxel size: 3.0 3.0 3.0 mm mm mm; (resel = 40.23 voxels)
