# Supplementary material for: Arterial spin labeling versus BOLD in direct challenge and drug-task interaction pharmacological fMRI
Source: PeerJ. 2014 Dec 11;2:e687. doi: 10.7717/peerj.687 (PMC4266850; doi:10.7717/peerj.687)
Supplement: Figure S12 — First page shows no statistically significant activation clusters and second page shows no statistically significant deactivation clusters. [file peerj-02-687-s018.pdf]

BOLD 2backxSYNxLDopa increases 60 mg only

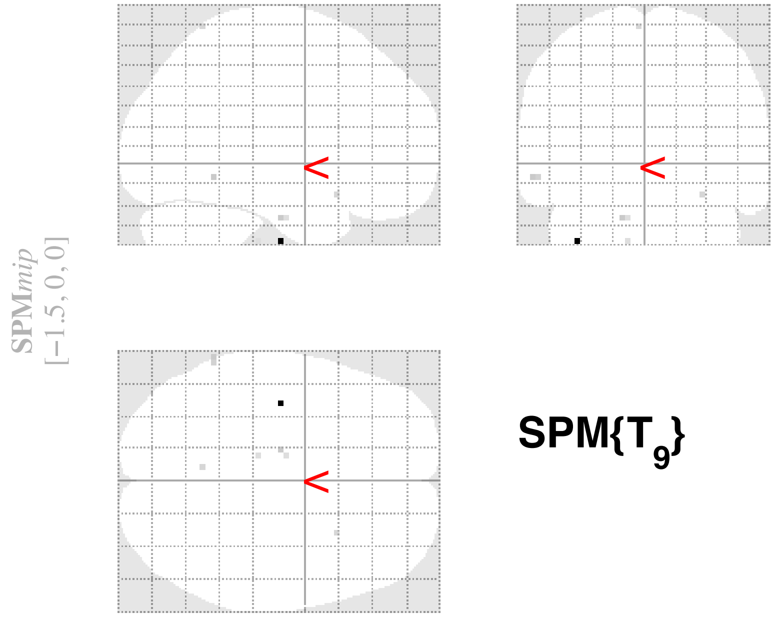

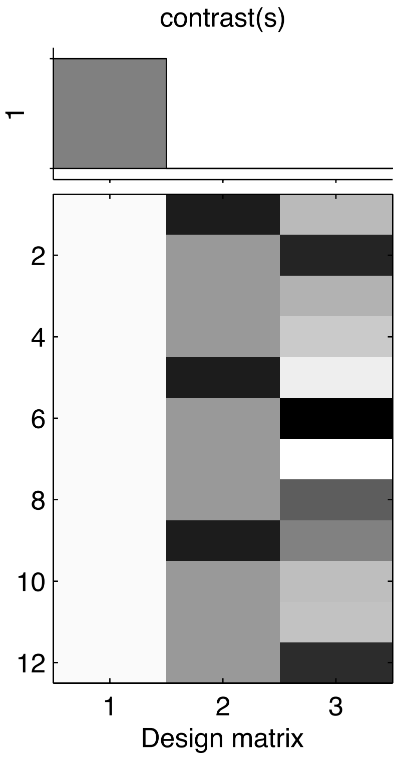

SPM{T_9}

SPMresults: ./bold_2backxSYNxLDopa_60_only
Height threshold T = 4.296806 {p<0.001 (unc.)}
Extent threshold k = 0 voxels

Statistics: *p−values adjusted for search volume*

set−level		cluster−level				peak−level					mm mm mm
p	c	$p_{FWE-corr}$	$q_{FDR-corr}$	k_E	p_{uncorr}	$p_{FWE-corr}$	$q_{FDR-corr}$	T	($Z_{\equiv}$)	p_{uncorr}	
1.000	6	1.000	0.390	1	0.390	0.999	0.890	6.83	3.96	0.000	−38 −15 −42
		1.000	0.390	2	0.224	1.000	0.921	4.60	3.22	0.001	−62 −51 −9
		1.000	0.390	2	0.224	1.000	0.921	4.60	3.22	0.001	−14 −15 −30
		1.000	0.390	1	0.390	1.000	0.921	4.50	3.18	0.001	−4 −57 69
		1.000	0.390	1	0.390	1.000	0.921	4.48	3.17	0.001	28 15 −18
		1.000	0.390	1	0.390	1.000	0.921	4.40	3.14	0.001	−10 −27 −42

table shows 3 local maxima more than 8.0mm apart

Height threshold: T = 4.30, p = 0.001 (1.000)
Extent threshold: k = 0 voxels
Expected voxels per cluster, <k> = 1.455
Expected number of clusters, <c> = 44.20
FWEp: 11.012, FDRp: Inf, FWEc: Inf, FDRc: Inf

Degrees of freedom = [1.0, 9.0]
FWHM = 9.9 10.1 8.2 mm mm mm; 3.3 3.4 2.7 {voxels}
Volume: 1692981 = 62703 voxels = 1876.3 resels
Voxel size: 3.0 3.0 3.0 mm mm mm; (resel = 30.25 voxels)

BOLD 2backxSYNxLDopa decreases 60 mg only

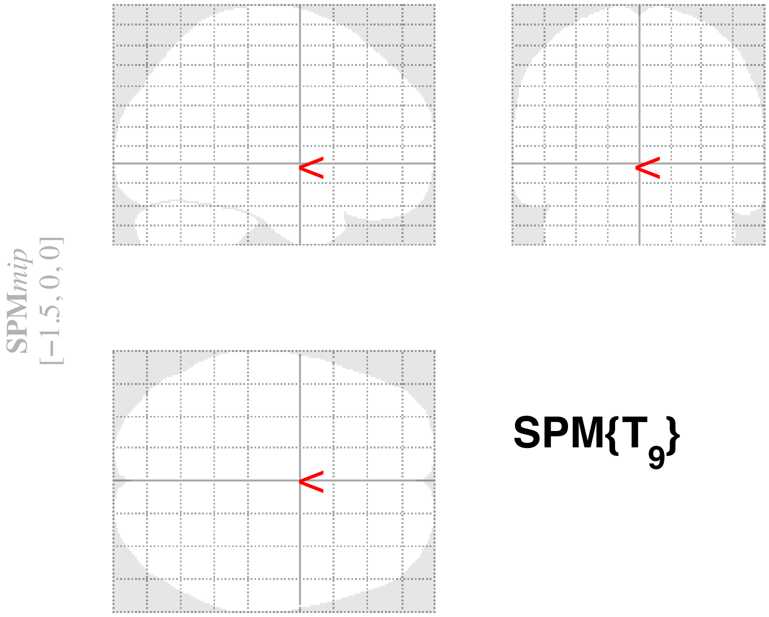

SPM{T_9}

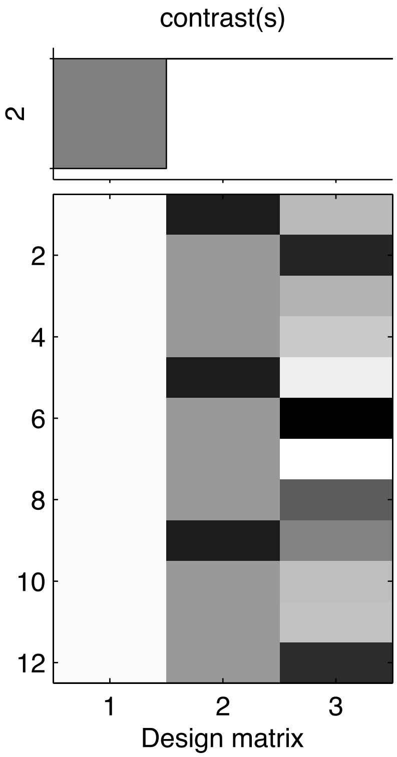

SPMresults: /bold_2backxSYNxLDopa_60_only
Height threshold T = 4.296806 {p<0.001 (unc.)}
Extent threshold k = 0 voxels

Statistics: ***p−values adjusted for search volume***

set−level		cluster−level				peak−level					mm mm mm
p	c	$p_{FWE-corr}$	$q_{FDR-corr}$	k_E	p_{uncorr}	$p_{FWE-corr}$	$q_{FDR-corr}$	T	($Z_{\equiv}$)	p_{uncorr}	

no suprathreshold clusters

table shows 3 local maxima more than 8.0mm apart

Height threshold: T = 4.30, p = 0.001 (1.000)
Extent threshold: k = 0 voxels
Expected voxels per cluster, <k> = 1.455
Expected number of clusters, <c> = 44.20
FWEp: 11.012, FDRp: Inf, FWEc: Inf, FDRc: Inf

Degrees of freedom = [1.0, 9.0]
FWHM = 9.9 10.1 8.2 mm mm mm; 3.3 3.4 2.7 {voxels}
Volume: 1692981 = 62703 voxels = 1876.3 resels
Voxel size: 3.0 3.0 3.0 mm mm mm; (resel = 30.25 voxels)
